# RNA helicases, DDX5 and DDX17, facilitate lytic reactivation of gammaherpesviruses

**DOI:** 10.1371/journal.ppat.1013009

**Published:** 2025-04-21

**Authors:** Praneet Kaur Sandhu, Blossom Damania

**Affiliations:** 1 Lineberger Comprehensive Cancer Center, University of North Carolina at Chapel Hill, Chapel Hill, North Carolina, United States of America; 2 Department of Microbiology and Immunology, University of North Carolina at Chapel Hill, Chapel Hill, North Carolina, United States of America; National Cancer Institute, UNITED STATES OF AMERICA

## Abstract

Human gammaherpesviruses comprise of Kaposi’s sarcoma-associated herpesvirus (KSHV) and Epstein-Barr virus (EBV), and are oncogenic viruses that cause life-long infections. The gammaherpesviruses utilize an extensive virus-host interaction network for facilitating viral replication, whereby virus-encoded proteins modulate host processes. Thus, identifying targets of viral proteins that aid in gammaherpesviral replication will help develop therapies to combat these viruses. We identified that host proteins DDX5 and DDX17 interact with gammaherpesviral protein kinases, KSHV-encoded vPK and EBV-encoded BGLF4. We found that DDX5 and DDX17 are required for gammaherpesviral lytic reactivation and loss of both DDX5 and DDX17 decreased KSHV and EBV lytic reactivation. Depletion of DDX5 and DDX17 lowered the transcription of KSHV RTA, the key viral gene that drives the lytic replication cascade, due to reduced occupancy of Brg1, a chromatin remodeler, at the RTA promoter. Consequently, inhibition of Brg1 decreased gammaherpesviral lytic reactivation. Here we demonstrate how gammaherpesviruses hijack the function of two host proteins to promote their lytic replication cycle.

## Author summary

Human gammaherpesviruses, KSHV and EBV, cause various epithelial, endothelial and B cell cancers, among others. These cancer-causing viruses utilize host proteins for virus replication and infection. Identifying which host proteins are required by the virus will enable the development of novel strategies to target these viruses, and prevent or cure diseases caused by them. Here, we found two host proteins, DDX5 and DDX17, that are required by KSHV and EBV for lytic reactivation, a phase of infection that is important for virus production. DDX5 and DDX17 promote virus infection by enhancing viral gene expression via recruitment of host protein Brg1, a chromatin remodeler. As such, inhibition of Brg1 decreases gammaherpesviral lytic reactivation. Our study highlights the interplay of viral and host proteins to control the viral lifecycle and identifies DDX5 and DDX17 as important regulators of gammaherpesviral lytic reactivation.

## Introduction

Gammaherpesviruses, namely Kaposi’s sarcoma-associated herpesvirus (KSHV) and Epstein-Barr virus (EBV), are oncogenic, double-stranded DNA viruses that cause persistent infections within the human host. KSHV causes the endothelial malignancy Kaposi’s sarcoma, as well as B cell disorders, primary effusion lymphoma (PEL) and multi-centric Castleman’s disease [1]. EBV-associated cancers include, but are not limited to, lymphomas, nasopharyngeal carcinoma and gastric cancer [2]. Gammaherpesviruses exhibit two distinct phases of infection (i) latency and (ii) lytic phase, and both lytic and latent proteins expressed by gammaherpesviruses contribute to the various diseases caused by the viruses. Moreover, lytic reactivation generates progeny virions that propagate infection in new hosts. Thus, understanding how lytic reactivation is regulated is important to control gammaherpesviral spread.

Initiation of the gammaherpesvirus lytic cycle requires expression of the immediate early (IE) lytic viral proteins. KSHV lytic reactivation is induced by the expression of replication and transcription transactivator, RTA [3,4]. On the other hand, EBV lytic reactivation is driven by the concerted action of two viral proteins- RTA (or R) and ZTA (Z or Zebra) [5]. The RTA protein of KSHV induces transcription by binding to RTA-responsive elements (RREs) and auto-regulates its own promoter [6]. ZTA and RTA recognize and bind distinct DNA elements within gene promoters and they can also regulate expression of each other by binding to their respective promoters [2,7]. Lytic reactivation causes restructuring of the promoter region of the IE lytic viral genes to allow for robust transcription. As such, there is an increase in transcription-activating histone modifications, deposited by various host chromatin modulators at the IE gene promoters during lytic reactivation [3,8–12]. In PEL, lytic reactivation is marked by nucleosome remodeling at the KSHV RTA promoter [13]. In addition, there is an increase in histone acetylation, and a decrease in histone methylation (H3K27me3) and phosphorylation at the RTA promoter during lytic reactivation [3,8,13,14]. Similarly, EBV also exhibits a transcription-promoting pattern of chromatin modifications at the RTA and ZTA promoter [10–12,15]. There is presence of repressive histone marks during latency, and both RTA and ZTA promoters exhibited histone acetylation after lytic induction [10,11,15]. Thus, transcription from the lytic cycle-inducing viral promoters during lytic reactivation is controlled by binding of host factors to induce chromatin remodeling and enhance chromatin accessibility to make the promoters transcription-permissive.

Herpesviruses encode for conserved herpesvirus protein kinases (CHPKs), which are important for viral infection. The gammaherpesviral protein kinases are most homologous in the CHPK family, and include viral serine/threonine protein kinase (vPK), encoded by ORF36 of KSHV, and EBV-encoded BGLF4. As a protein kinase, vPK has many viral and cellular targets and modulates various cellular processes. vPK augments cellular protein synthesis and proliferation, and activates the c-Jun N-terminal kinase (JNK) pathway [16,17]. vPK also regulates the DNA repair pathway and inhibits the production of type I interferon, similar to BGLF4 [18–20]. Interestingly, transgenic mice overexpressing vPK had increased incidence of B cell lymphomas, highlighting the importance of vPK in KSHV-associated pathogenesis [21]. Another important role that vPK plays during the viral lifecycle is to promote lytic replication by phosphorylating a repressive transcription factor KAP1 and causing its dissociation from the viral genome [22]. The EBV homolog of vPK, BGLF4, shares common functions with vPK [23]. vPK and BGLF4 phosphorylate common host targets like the nuclear lamina and retinoblastoma proteins [23]. BGLF4 is packaged as part of the virion and can also inhibit the IRF3 pathway, similar to vPK [19,24–26]. In addition, both vPK and BGLF4 phosphorylate viral proteins to promote the viral lytic cycle [24,26–30]. Importantly, depletion of vPK or BGLF4 during the lytic phase of virus reduces the production of infectious virions [28,31]. Given the importance of viral protein kinases in the gammaherpesviral lifecycle, it is important to fully explore the interactions of viral protein kinases with the host substrates and determine how these interactions influence viral replication.

DDX5 and DDX17 are members of the DDX/DHX family of RNA helicases, the largest family of RNA helicases that is highly conserved across species [32]. Interestingly, DDX5 and DDX17 proteins are paralogs within this family and exhibit high sequence similarity in the core region with divergence in sequence at the N and C termini [33,34]. In addition, DDX5 and DDX17 form homodimers in the cell but can also heterodimerize with each other [35]. These proteins can localize to the nucleus and cytoplasm with distinct functions in each compartment [36,37]. They are commonly found to be overexpressed in cancer and enhance cell proliferation of cancer cells [33,34]. As RNA-binding proteins, DDX5 and DDX17 have extensive roles in RNA metabolism within the cell. These proteins are involved in ribosome biogenesis and miRNA maturation, and can regulate splicing, RNA export, storage and decay [32,38]. Transcriptional regulation is another function common to both DDX5 and DDX17 as these proteins can recruit transcription factors and chromatin-modifying proteins to influence gene expression [39–42]. Of note, DDX5 and DDX17 can exhibit functional overlap [33,34]. DDX5 and DDX17 also modulate infection of RNA viruses owing to their RNA-binding properties. DDX5 and DDX17 promote influenza, HIV-1 and Sindbis infection, and singularly have proviral or antiviral roles in other RNA or DNA virus infections [43–48]. However, their function in herpesvirus infections is poorly understood.

We sought to characterize the roles of DDX5 and DDX17 during gammaherpesviral lytic reactivation. We show that both DDX5 and DDX17 interact with gammaherpesviral protein kinases, KSHV-encoded vPK and EBV-encoded BGLF4. Importantly, we demonstrate that DDX5 and DDX17 are required for lytic reactivation of gammaherpesviruses. During KSHV lytic reactivation, the DDX proteins exhibit functional redundancy and engage Brg1, a SWI-SNF complex chromatin remodeler, at the promoter of RTA, the viral lytic switch gene. Inhibition of Brg1 reduced lytic reactivation for both KSHV and EBV. Thus, we identify DDX5 and DDX17 as interacting partners of viral protein kinases and highlight their roles in the modulation of gammaherpesviral lytic reactivation via Brg1.

## Results

### DDX5 and DDX17 interact with gammaherpesviral protein kinases

**Fig 1:**
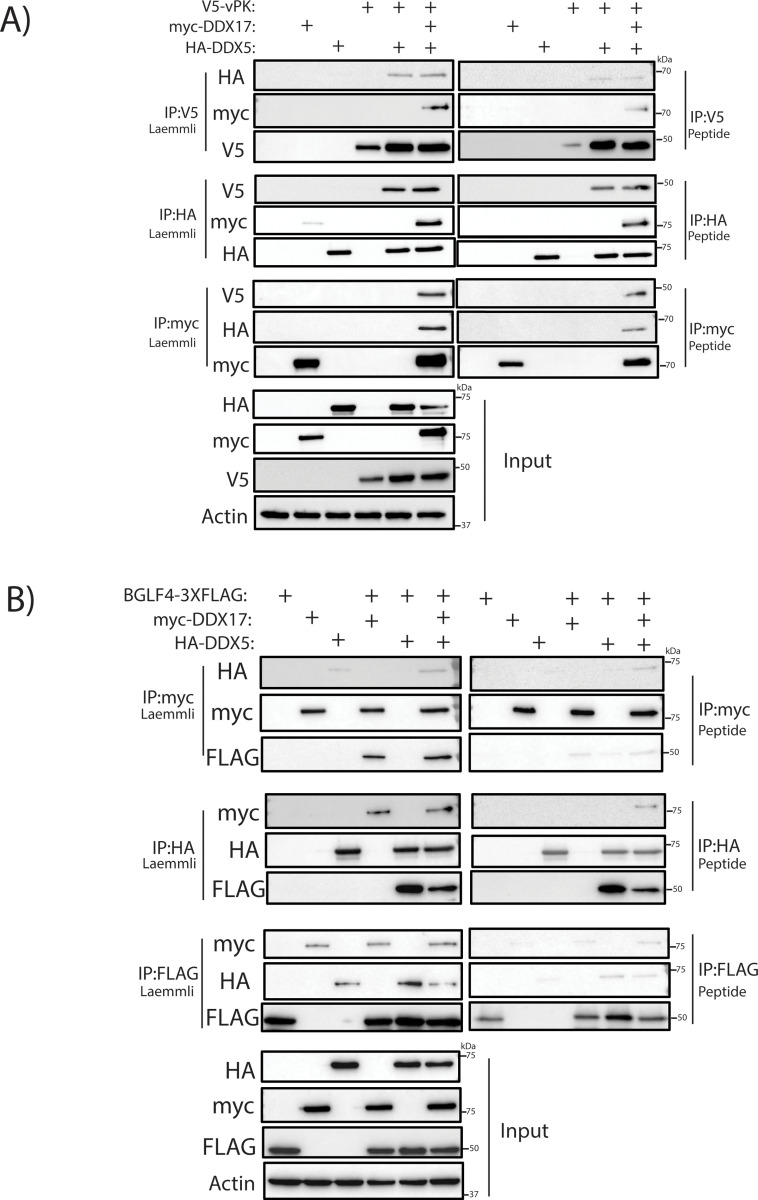
DDX5 and DDX17 interact with KSHV vPK and EBV BGLF4. Western blot showing immunoprecipitation of A) myc-DDX17, HA-DDX5 and V5-vPK using anti-V5, anti-HA or anti-myc agarose beads from transfected 293T cell lysates harvested at 72 h post transfection and B) myc-DDX17, HA-DDX5 and BGLF4-3XFLAG using anti-FLAG, anti-HA or anti-myc agarose beads from transfected 293T cell lysates harvested at 48 h post transfection. Immunoprecipitations were eluted using Laemmli sample loading buffer and competitive peptide. Input shows actin as loading control.

To identify host binding partners of vPK, we previously performed immunoprecipitation (IP) for vPK followed by mass-spectrometry (MS). The IP-MS identified DDX17 as one of the top binding partners of vPK [49]. Additionally, the paralog of DDX17, DDX5, was also present in the list of vPK-interacting proteins. To confirm the IP-MS results, we performed co-immunoprecipitation (co-IP) experiments in HEK293T cells using tagged DDX5, DDX17 and vPK. When we co-transfected myc-tagged DDX17 and V5-tagged vPK and immunoprecipitated V5-vPK, we found the presence of myc-DDX17 in the complex (S1A Fig). Similarly, immunoprecipitation of myc-DDX17 pulled down V5-vPK (S1A Fig). The interaction was confirmed using both Laemmli sample buffer and competitive peptide elutions for the co-IP to ensure high stringency for protein-protein interactions (S1A Fig). Next, we performed similar experiments for myc-tagged DDX5. Like DDX17, myc-DDX5 was pulled down when V5-vPK was immunoprecipitated (S1B Fig). We also conducted the reverse co-IPs where V5-vPK was pulled down when myc-DDX5 or myc-DDX17 were immunoprecipitated (S1A, B Fig). Interestingly, we also observed an increase in myc-DDX17 and V5-vPK expression when co-expressed together (S1A Fig). Similarly, V5-vPK expression was also increased when co-expressed with myc-DDX5 (S1B Fig). Since DDX5 and DDX17 can heterodimerize, we wanted to determine if DDX5 and DDX17 were present as a complex with vPK. To test this, we modified the tag on DDX5 from myc to HA using site-directed mutagenesis. Then, we co-expressed myc-DDX17, HA-DDX5 and V5-vPK together. We confirmed that the change of the tag from myc to HA did not affect the ability of DDX5 to interact with vPK (Fig 1A). Importantly, when V5-vPK was immunoprecipitated, myc-DDX17 and HA-DDX5 were both pulled down (Fig 1A). We further confirmed this by performing reverse co-IP experiments using HA and myc beads where the HA-DDX5 IP pulled down V5-vPK and myc-DDX17, and the myc-DDX17 IP pulled down V5-vPK and HA-DDX5 (Fig 1A). The specificity of these co-immunoprecipitations was additionally confirmed by conducting peptide elutions where the pulldown results were similar to the Laemmli elutions (Figs 1A and S1B). We also tested whether vPK could interact with DDX5 and DDX17 during lytic reactivation. We transfected V5-vPK into iSLK.219 and 293T.219 cells, two epithelial cell lines latently infected with KSHV, and induced lytic reactivation. Using lysate derived from reactivated cells, IP of V5-vPK pulled down endogenous DDX5 and DDX17 (S1C Fig). Thus, vPK interacts with DDX5 and DDX17 individually, and as DDX5 and DDX17 can heterodimerize, the three proteins could be part of a complex.

Since DDX5 and DDX17 could bind to vPK, we wondered if these proteins also interacted with the EBV homolog of vPK, BGLF4. We overexpressed 3X-FLAG tagged BGLF4 with HA-DDX5 and myc-DDX17 in 293T cells and performed co-IP experiments. We found that co-IP of myc-DDX17 pulled down both HA-DDX5 and BGLF4-3XFLAG (Fig 1B). Similarly, immunoprecipitation of HA-DDX5 or BGLF4-3XFLAG pulled down the DDX proteins in complex with BGLF4 in the reverse coIPs (Fig 1B). As proteins can sometimes non-specifically bind to the beads, we also performed peptide elutions to confirm our coIP results (Fig 1A, B). Thus, DDX5 and DDX17 interact with gammaherpesviral protein kinases vPK and BGLF4, and these proteins could be part of a complex.

### DDX5 and DDX17 promote lytic reactivation of KSHV in iSLK.219 cells

**Fig 2:**
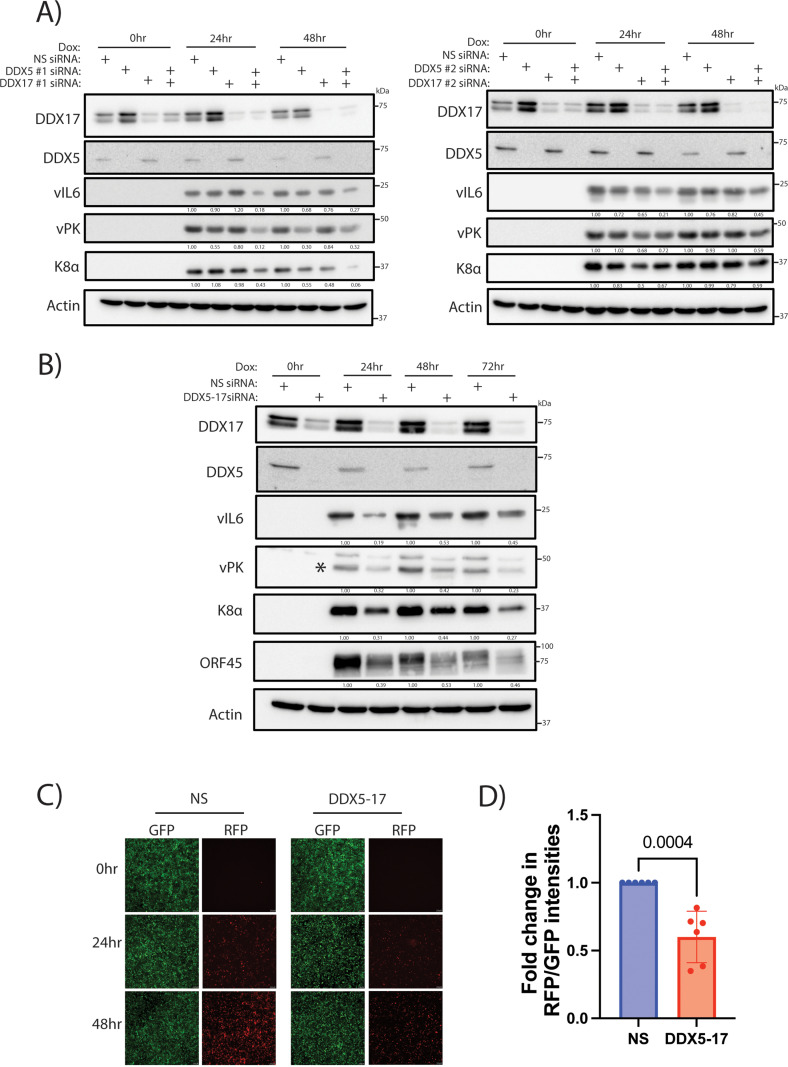
DDX5 and DDX17 are required for KSHV lytic reactivation. iSLK.219 cells transfected with non-specific (NS), DDX5, DDX17 siRNAs and treated with doxycycline (2μg/ml) 48 h post transfection. A) Western blot of vIL6, vPK, K8a and host proteins DDX5, DDX17 and actin (loading control) at 48 h post transfection (no doxycycline, 0 h), 24 h and 48 h post doxycycline addition with siRNAs targeting DDX5 and DDX17 singly or in combination. Two different siRNAs targeting DDX5 and DDX17 are depicted in the figure. B) Western blot analysis of vIL6, vPK, K8a, ORF45 and host proteins DDX5, DDX17 and actin (loading control) at 48 h post transfection (no doxycycline, 0 h), 24 h, 48 h and 72 h post doxycycline addition with a single siRNA targeting both DDX5 and DDX17. C) Images for GFP and RFP from iSLK.219 cells transfected with NS or DDX5-17 siRNA, and treated with doxycycline (2μg/ml) at 0 h, 24 h and 48 h post doxycycline treatment. D) Quantification of GFP and RFP fluorescence intensities from C to calculate RFP to GFP ratio. Fold change was normalized to NS at 48 h post doxycycline addition. p values are the result of Student’s t tests and error bars indicate the standard deviation from three or more independent replicates.

Since DDX5 and DDX17 interact with vPK (Figs 1A and S1A–C), we wanted to determine whether the DDX proteins were required for KSHV lytic reactivation. We utilized short, interfering RNA (siRNA) to deplete DDX5 and DDX17 in iSLK.219 cells, an epithelial cell line latently infected with recombinant KSHV expressing green fluorescent protein (GFP) constitutively and PAN promoter-driven red fluorescent protein (RFP) upon lytic reactivation [50,51]. In iSLK.219 cells, lytic reactivation can be induced by the addition of doxycycline (dox) as these cells express RTA driven by a doxycycline-inducible promoter [50,51]. Using two different siRNA sequences to target DDX5 and DDX17 singly, we observed that transfection of siRNA targeting only DDX5 or DDX17 in iSLK.219 cells did not change expression of KSHV viral proteins, viral interleukin 6 (vIL6), vPK, and K8α compared to the non-specific (NS) control at 24 and 48 h post reactivation (Fig 2A). However, the depletion of both DDX5 and DDX17 reduced expression of viral proteins vIL6, K8α, and vPK in comparison to the NS control (Fig 2A). This result demonstrates that DDX5 and DDX17 exhibit functional redundancy during KSHV lytic reactivation, and that both proteins are required for optimal reactivation of the virus in iSLK.219 cells. Next, we used a single siRNA construct that has been shown previously to target both DDX5 and DDX17 [39]. Using the single siRNA targeting both DDX5 and DDX17 (DDX5-17), we observed decreased expression of viral proteins vIL6, K8α, vPK, and ORF45 compared to the NS siRNA control over the course of lytic reactivation (Fig 2B). As the iSLK.219 cells express RFP only upon lytic reactivation, imaging iSLK.219 cells for both GFP and RFP can serve as a readout for viral lytic reactivation. We imaged reactivated iSLK.219 cells transfected with NS or DDX5-17 targeting siRNA and observed fewer RFP positive cells when the DDX proteins were depleted while the NS siRNA transfected iSLK.219 cells had a higher number of RFP positive cells (Fig 2C). The siRNA transfected cells were imaged for GFP and RFP, and the intensities of GFP and RFP were used to compute the RFP/GFP ratio. The RFP/GFP ratio was lower for the DDX5-17 siRNA transfected cells compared to the NS siRNA (Fig 2D). In addition, we depleted DDX5 and DDX17 using siRNA in iSLK-RTA cells, an iSLK cell line that expresses doxycycline-inducible RTA but lacks the presence of KSHV [50]. We found that the doxycycline-dependent expression of RTA does not change with DDX5 and DDX17 knockdown (S2A Fig). This shows that the reduced lytic reactivation of KSHV in the DDX5-17 depleted iSLK.219 cells is not due to a decrease in doxycycline-driven RTA expression (S2A Fig). Importantly, we found that the depletion of DDX5 and DDX17 did not reduce the number of live cells in the iSLK.219 cell line (S2B Fig). Thus, DDX5 and DDX17 are important for KSHV lytic reactivation.

**Fig 3:**
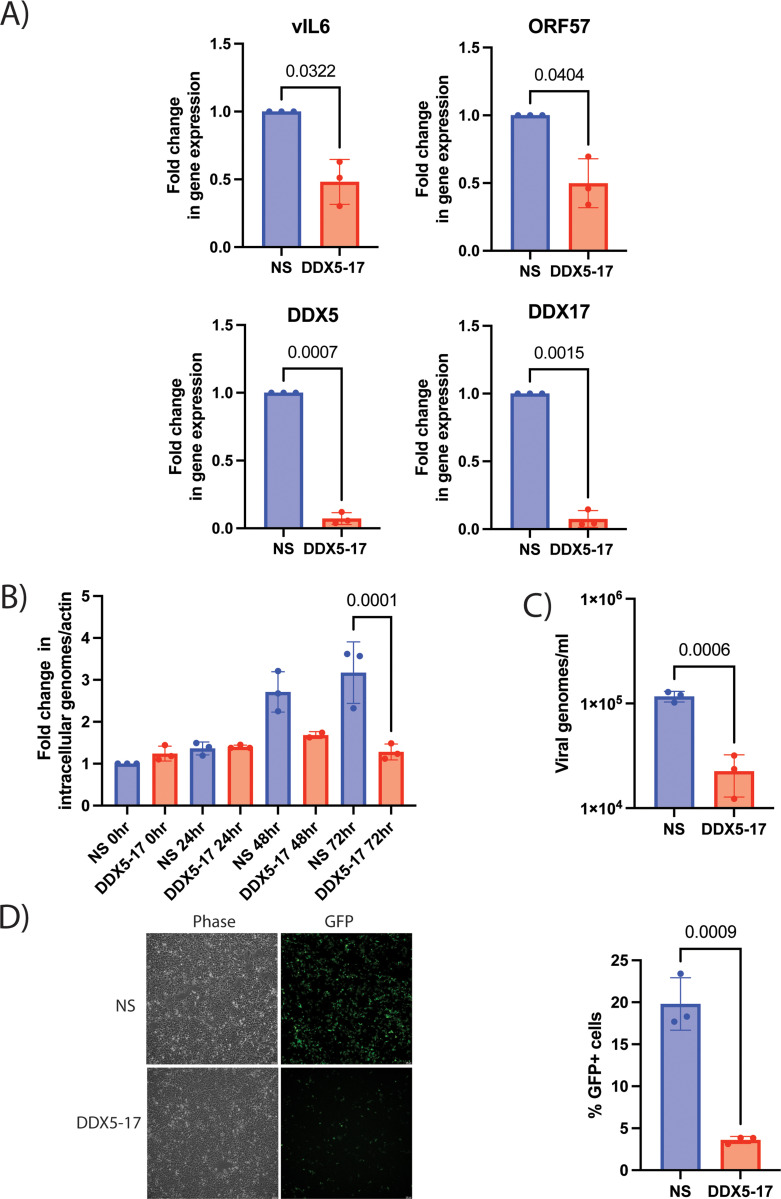
DDX5 and DDX17 decrease viral gene expression and virion production during KSHV lytic reactivation. iSLK.219 cells transfected with NS or DDX5-17 siRNA and treated with doxycycline (2μg/ml) 48 h post transfection. A) RT-qPCR analysis of viral genes vIL6, ORF57 and host genes DDX5, DDX17 with actin as loading control (normalized to NS siRNA) at 24 h after doxycycline addition. B) Quantification of viral genomes that are cell-associated (relative to actin, normalized to NS 0 h) or C) in the supernatant of iSLK.219 cells transfected with NS or DDX5-17 siRNA and treated with doxycycline (2μg/ml). Viral genomes were quantified by qPCR using primers for the viral gene, ORF39. D) Phase contrast and GFP images of 293 cells subjected to the supernatant transfer assay at 48h post transfer. Supernatant was transferred from reactivated iSLK.219 cells transfected with NS, DDX5-17 siRNA and treated with doxycycline (2μg/ml) at 72 h post doxycycline onto naïve, uninfected 293 cells. Quantification of virion infectivity by supernatant transfer assay at 48 h post transfer. p values are the result of Student’s t tests and error bars indicate the standard deviation from three independent replicates.

To understand how DDX5 and DDX17 regulate KSHV lytic reactivation, we assessed transcription of KSHV genes vIL6 (early/latent), PAN, ORF16, ORF57, vPK (immediate-early or early), K8.1, ORF22, ORF42, ORF52 (late). We found reduced transcription of vIL6, ORF57, ORF16, PAN, vPK, ORF52 and ORF42 post reactivation when the DDX proteins were knocked down (Figs 3A and S3A). We also confirmed knockdown of DDX5 and DDX17 and verified induction of lytic reactivation by measuring viral transcripts in latent and reactivated cells by qRT-PCR (Figs 3A and S3B). However, there was no significant decrease in the transcription of late genes, K8.1 and ORF22 (S3A Fig). As DDX5 and DDX17 can regulate ribosomal RNA (rRNA) synthesis, we next checked whether rRNA synthesis was affected by the DDX5-17 depletion [52]. We found that rRNA transcription was not decreased upon DDX5-17 depletion in iSLK.219 cells, indicating that the siRNA knockdown of DDX5 and DDX17 reduced viral gene transcription and did not alter global cellular transcription (S4 Fig). Next, we evaluated the production of virions upon DDX5-17 knockdown in iSLK.219 cells. Using qPCR, we quantified viral DNA within the cell (viral DNA replication) and in the supernatant. We found a significant reduction in both viral DNA replication and viral DNA from virions in the supernatant at 72 h post reactivation (Fig 3B, C). We also looked at the infectivity of the virions produced by iSLK.219 cells when DDX5 and DDX17 were knocked down. We transferred virion-containing supernatant from reactivated iSLK.219 cells transfected with NS or DDX5-17 siRNA onto uninfected 293 cells and measured GFP positive cells at 48 h post supernatant transfer. We observed fewer GFP positive cells in the DDX5-17 siRNA supernatant-treated 293 cells compared to the NS siRNA control (Fig 3D). Hence, DDX5 and DDX17 proteins promote KSHV lytic reactivation in iSLK.219 cells.

### DDX5 and DDX17 promote KSHV lytic reactivation in BCBL1 cells and KSHV primary infection in HUVEC

**Fig 4:**
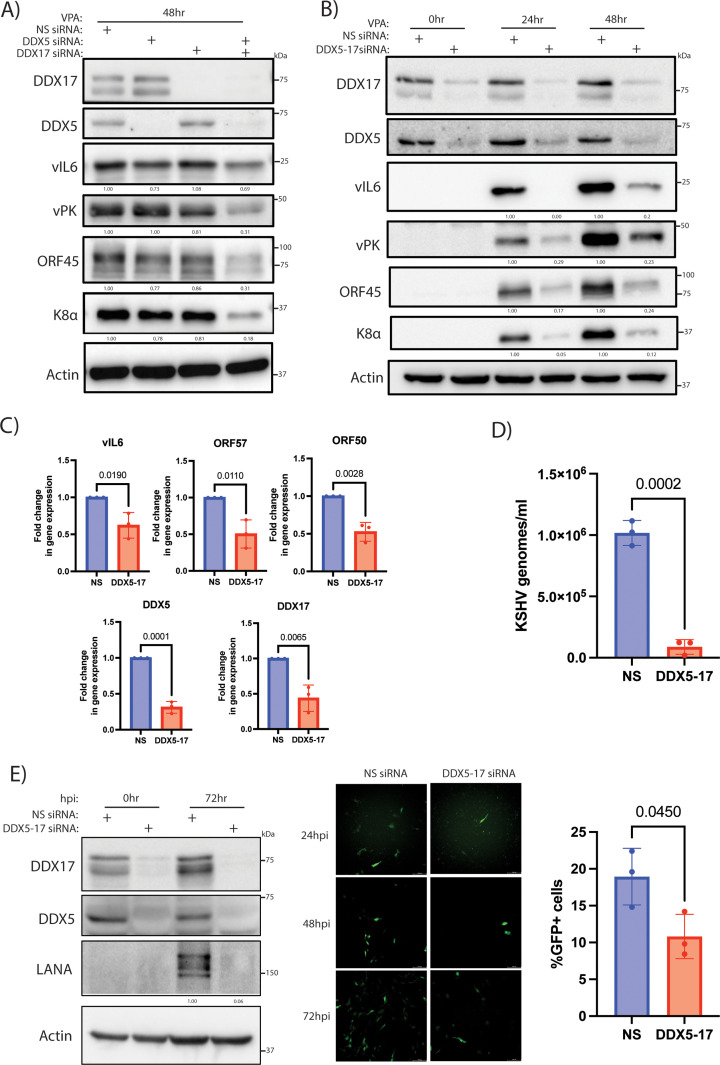
DDX5 and DDX17 are required for KSHV lytic reactivation in BCBL1 cells and primary infection in endothelial cells. Western blot analysis for vIL6, vPK, K8a, ORF45 and host proteins DDX5, DDX17 and actin (loading control) from BCBL1 cells transfected with A) NS siRNA, single DDX5 or DDX17 siRNAs and B) NS, dual DDX5-17 siRNAs at 0 h, 24 h at 48 h post valproic acid (VPA) treatment. C) RT-qPCR analysis of viral genes vIL6, ORF57, and ORF50 and host genes DDX5, DDX17 with actin as loading control (normalized to NS siRNA) at 24 h after VPA addition. D) Quantification of viral genomes in the supernatant by qPCR using primers for viral gene, ORF39, from BCBL1 cells transfected with NS or DDX5-17 siRNA and treated with 1mM VPA. E) Western blot for viral protein, LANA, and host proteins, DDX17, DDX5, with actin as loading control at 72 h post infection for HUVEC cells treated with NS or DDX5-17 siRNAs and infected with KSHV (isolated from iSLK.219 cells). Images for GFP from KSHV-infected HUVEC treated with NS or DDX5-17 siRNAs at 24 h, 48 h and 72 hpi and quantification of GFP positive cells using flow cytometry at 72 hpi. p values are the result of Student’s t tests and error bars indicate the standard deviation from three independent replicates.

PEL is an aggressive B cell lymphoma with KSHV as the etiological agent. As DDX5 and DDX17 knockdown caused decreased KSHV lytic reactivation in iSLK.219 cells, we next evaluated if the naturally infected PEL cell line, BCBL1, also required DDX5 and DDX17 for lytic reactivation. We transfected DDX5 and DDX17 specific siRNA to deplete these proteins in BCBL1 cells and used valproic acid to induce lytic reactivation of KSHV. Upon knockdown of DDX5 or DDX17 alone, the expression of viral proteins vIL6, vPK, ORF45, and K8α was unchanged relative to NS control (Fig 4A). However, knockdown of both DDX5 and DDX17 reduced the expression of these viral proteins (Fig 4A). Also, the knockdown of DDX5 and DDX17 did not cause a decrease in cell viability in BCBL1 cells (S2C Fig). In addition, using the single siRNA to target both DDX proteins, we confirmed decreased expression of the lytic proteins vIL6, vPK, ORF45 and K8α in the DDX-depleted BCBL1 cells (Fig 4B). Additionally, there was a decrease in transcription of KSHV genes vIL6, ORF57, PAN, and vPK upon knockdown of DDX5 and DDX17 while late genes K8.1, ORF22, ORF52 and ORF42 were not significantly reduced (Figs 4C and S5A). Importantly, there was less transcription of ORF50, the viral gene that encodes for the key lytic switch protein RTA, in the DDX5-17 siRNA-transfected BCBL1 cells (Fig 4C). Lytic induction was also verified by measuring viral transcripts in latent and reactivated BCBL1 cells (S5B Fig). In addition to a decrease in viral gene transcription and protein expression, the depletion of DDX5 and DDX17 also led to a significant reduction in the number of virions produced in the supernatant (Fig 4D). Thus, DDX5 and DDX17 are required for KSHV reactivation in PEL cells.

As Kaposi’s sarcoma is an endothelial cancer, we next wanted to check if the DDX proteins were important for infection in the endothelial cells. KSHV infects endothelial cells with a short burst of lytic gene expression followed by establishment of latency [53]. Since viral gene transcription was lower upon DDX5/17 knockdown, we hypothesized that DDX5 and DDX17 are important for primary infection. To test this, we infected immortalized human umbilical vein endothelial cells (HUVEC) transfected with NS or DDX5-17 siRNA using KSHV isolated from reactivated iSLK.219 cells. We performed Western blot analysis to probe for LANA as a marker for infection. Also, the infection was monitored by fluorescent imaging of the cells to track GFP expression and quantification of GFP-positive, KSHV-infected HUVEC by flow cytometry. Knockdown of DDX5 and DDX17 reduced KSHV infection of HUVEC as seen by decreased expression of LANA and fewer GFP-positive cells in the DDX5-17 siRNA treated HUVEC (Fig 4E). We also found no difference in viral entry for NS vs DDX5-17 HUVEC at 4hpi (S5C Fig). Hence, DDX5 and DDX17 aid in primary infection of KSHV in endothelial cells.

### DDX5 and DDX17 recruit Brg1 to the RTA promoter

To determine how DDX5 and DDX17 modulate KSHV lytic reactivation and infection, we wanted to assess the localization of DDX5 and DDX17 and determine if their localization is altered during lytic reactivation. Immunofluorescence imaging of DDX5 and DDX17 in iSLK.219 cells revealed that both DDX proteins are nuclear in localization during latency (S6A, B Fig). Importantly, both DDX5 and DDX17 remained in the nucleus after lytic reactivation as seen by the DDX5 and DDX17 nuclear signal in the RFP positive iSLK.219 cells (S6A, B Fig). We also determined localization of DDX5 and DDX17 during reactivation of PEL cells by using TREx-BCBL1-RTA, a BCBL1 cell line that contains doxycycline-inducible RTA expression to drive lytic reactivation. Using this cell line, we found that DDX5 and DDX17 remained nuclear upon lytic reactivation, similar to the iSLK.219 cells (S6C Fig). Using this cell line, we also stained for ORF59 to mark the replication and transcription compartments in the reactivating TREx-BCBL1-RTA cells. We observed that the DDX5 and DDX17 signal had overlap with the ORF59 signal (S6C,D Fig). Thus, DDX5 and DDX17 play a role in the nucleus upon lytic reactivation and maybe associated with the replication and transcription compartments.

**Fig 5:**
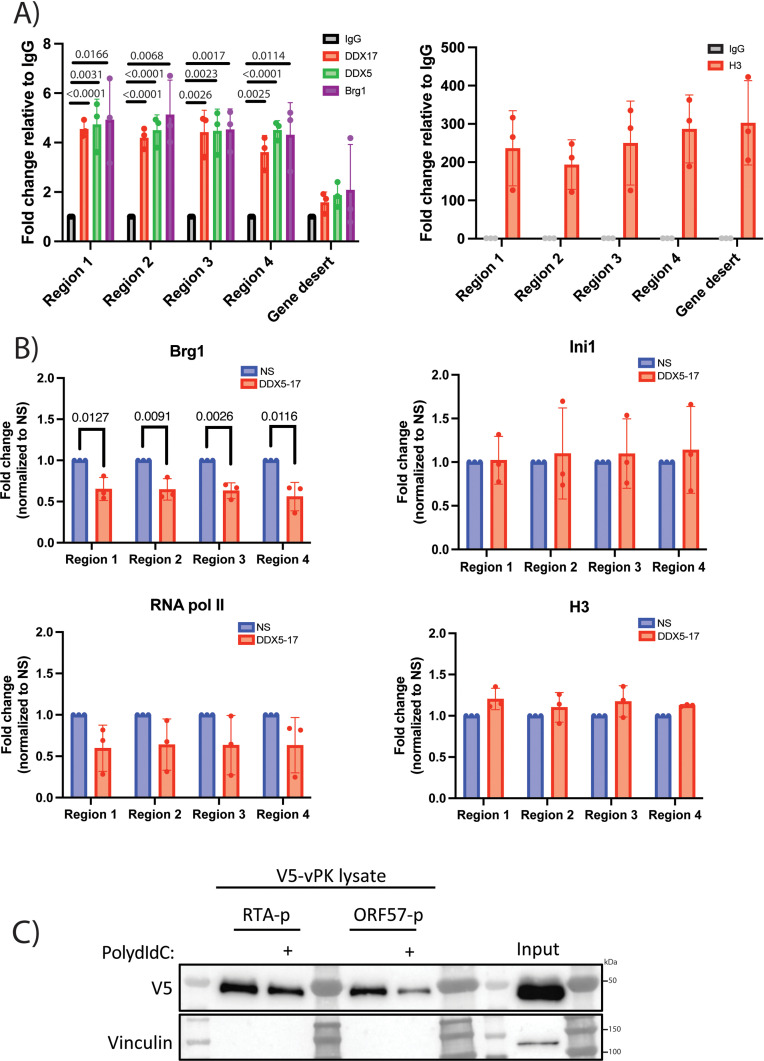
DDX5 and DDX17 bind to the RTA promoter and recruit Brg1. ChIP assay for A) DDX5, DDX17, Brg1, H3 or control rabbit or mouse IgG in iSLK.219 cells treated with doxycycline (2μg/ml) for 24 h followed by qPCR with primers targeting 4 regions within the viral RTA promoter and a primer set targeting a gene desert in the human genome as negative control and B) Brg1, Ini1, RNA pol II and H3 or control rabbit or mouse IgG in iSLK.219 cells transfected with NS or DDX5-17 siRNAs and treated with doxycycline (2μg/ml) at 24 h post doxycycline addition, normalized to NS. C) Western blot for V5 after DNA pulldown assay using biotinylated RTA and ORF57 promoters incubated with V5-vPK lysate in the presence or absence of poly dI:dC (20μg/ml) with vinculin as control. p values are the result of Student’s t tests and error bars indicate the standard deviation from three independent replicates.

DDX5 and DDX17 modulate transcription by recruiting transcription and chromatin remodeling machinery such as RNA polymerase II, TATA binding protein and Brg1, the catalytic subunit of the mammalian SWI/SNF complex [40]. Since the depletion of DDX5 and DDX17 reduced RTA transcription, we wondered if the recruitment of transcription or chromatin remodeling machinery was altered with the loss of DDX5 and DDX17 during lytic reactivation. We performed chromatin immunoprecipitation (ChIP) for DDX5 and DDX17 in latent and reactivated iSLK.219 cells at 24 h post reactivation followed by qPCR using primers targeting four different regions within the viral RTA promoter. As a control for the ChIP experiment, we also immunoprecipitated histone H3. DDX5 and DDX17 bound to the endogenous RTA promoter with approximately four-fold enrichment over the negative control IgG in iSLK.219 cells during lytic reactivation, and not at a gene desert within the human genome, demonstrating the specificity of DDX5 and DDX17 binding to the viral RTA promoter (Fig 5A). DDX5 and DDX17 showed some binding over control IgG at the RTA promoter during latency but their occupancy at the RTA promoter was higher during lytic reactivation (Figs 5A and S7A). Additionally, histone H3 was associated with the viral promoter and also with the gene desert as expected (Fig 5A). Thus, DDX5 and DDX17 occupied the viral RTA promoter during lytic reactivation of KSHV. Next, we performed ChIP-qPCR for members of the transcription machinery or SWI-SNF complex that bind to the RTA promoter. Brg1 and Ini1/SNF5 are components of the SWI/SNF complex for chromatin remodeling that are present at the RTA promoter along with recruitment of RNA pol II for transcription [13,54,55]. We found significant enrichment of Brg1 binding at the RTA promoter during lytic reactivation (Figs 5A and S7A). Additionally, we also observed binding of DDX5, DDX17 and Brg1 at the ORF57 promoter during lytic reactivation (S7B Fig). Interestingly, we observed decreased occupancy of Brg1, the ATPase subunit of the SWI/SNF complex, when DDX5 and DDX17 were depleted during lytic reactivation (Fig 5B). However, the presence of RNA pol II, Ini1/SNF5 and H3 at the RTA promoter was not significantly altered between iSLK.219 cells transfected with NS or DDX5-17 siRNAs (Fig 5B). Thus, DDX5 and DDX17 promote binding of Brg1 to the RTA promoter during KSHV lytic reactivation.

vPK has been reported to bind the promoters of viral genes ORF57 and K8α [26]. Since DDX5 and DDX17 interact with vPK and both the DDX proteins bind to the RTA promoter, we next wondered if vPK also bound to the RTA promoter. Since our vPK antibody is not ChIP-compatible, we performed a DNA pulldown assay using the RTA promoter as bait to see if vPK bound to it. We amplified the RTA and ORF57 promoters via PCR from DNA extracted from iSLK.219 cells using primers conjugated to biotin at the 5’ end. The biotin-labeled promoter DNA was incubated with streptavidin-conjugated magnetic Dynabeads to allow the biotinylated DNA probes to bind to the beads. Lysate obtained from 293T cells transfected with V5-vPK was then added to the promoter-bead complex and incubated overnight. To ensure binding specificity, we also prepared the same reaction with the addition of poly dI-dC to serve as a non-specific DNA competitor in excess. The following day, the beads were washed with RIPA buffer before addition of SDS-containing sample buffer for elution and the eluates were subjected to SDS-PAGE and Western blotting. We observed that V5-vPK bound to the positive control ORF57 promoter as previously reported [26] both in the presence or absence of poly dIdC (Fig 5C). Similar to ORF57, vPK also bound to the RTA promoter under both conditions (Fig 5C). We also did not see the presence of vinculin in the DNA pulldown conditions demonstrating the specificity of binding (Fig 5C). Next, we transfected V5-vPK into 293T.219 cells and performed ChIP for V5 at 24 h post reactivation. We found between 4 to 6 fold enrichment of V5-vPK at the RTA promoter and 10 fold enrichment at the ORF57 promoter (S7C Fig). We also conducted the promoter binding assay for the DDX proteins and Brg1 at both the RTA and ORF57 promoters. We found that Brg1 did not bind either promoter but the DDX proteins and vPK could bind both promoters in vitro (S7D Fig). Lastly, we depleted DDX5 and DDX17 using siRNA, transfected V5-vPK into 293T.219 cells and performed ChIP for V5 at 24h post reactivation. We found that three of the four regions for RTA promoter showed a decrease in V5-vPK binding as well as reduced occupancy at the ORF57 promoter (S7E Fig). Thus, vPK binds to the RTA promoter and may interact with the DDX proteins at the RTA promoter.

### Brg1 is required for KSHV lytic reactivation

**Fig 6:**
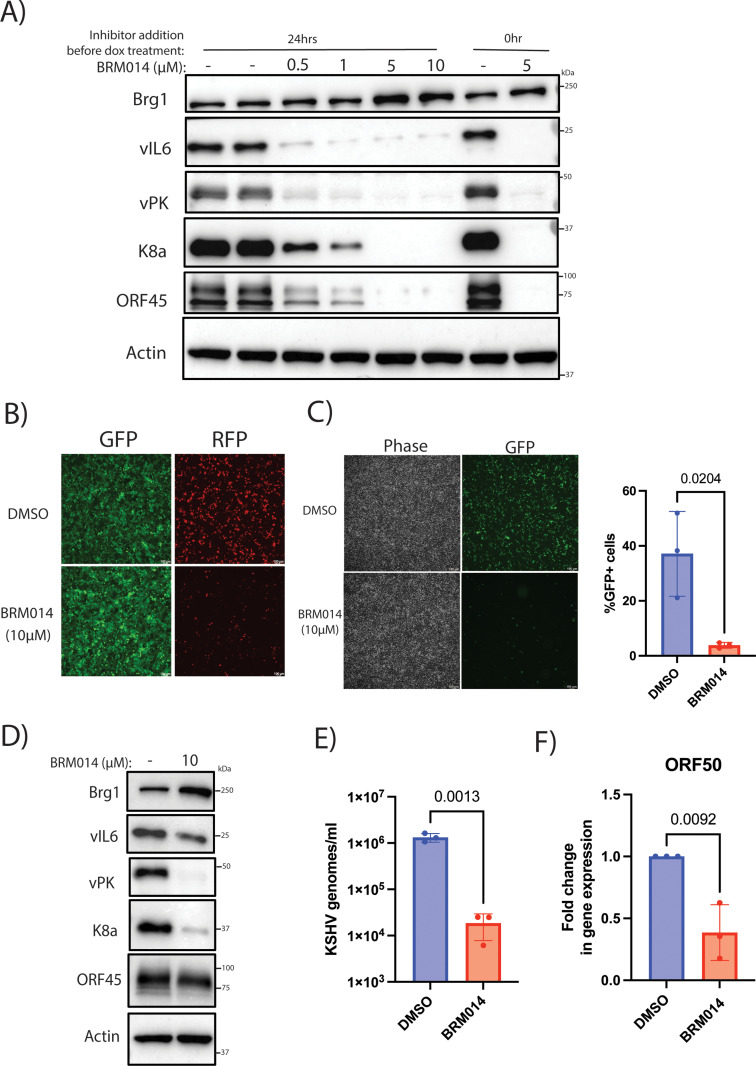
Brg1 inhibition reduces KSHV lytic reactivation. A) Western blot for proteins vIL6, vPK, K8a, ORF45, Brg1 and actin (loading control) from iSLK.219 cells treated with increasing concentrations of Brg1 inhibitor BRM014 (0.5μM, 1μM, 5μM and 10μM) or DMSO at 24 h pre-reactivation or BRM014 (5μM) at the time of reactivation, and treated with doxycycline (0.2μg/ml) for 48 h. B) Images for GFP and RFP from iSLK.219 cells treated with 10μM Brg1 inhibitor and treated with doxycycline (0.2μg/ml) at 48 h post reactivation as described above. C) Phase contrast and GFP images of 293 cells subjected to the supernatant transfer assay at 48h post transfer. Supernatant was transferred from reactivated iSLK219 cells treated with 10μM Brg1 inhibitor and doxycycline (0.2μg/ml) for 72 h and transferred onto naïve, uninfected 293 cells. Quantification of virion infectivity from supernatant transfer assay by flow cytometry at 48 h post transfer. D) Western blot analysis of viral proteins vIL6, vPK, K8a, ORF45 and host proteins Brg1, actin (loading control) at 48 h post reactivation in BCBL1 cells treated with 10μM Brg1 inhibitor for 24 h and reactivated with addition of 1mM valproic acid (VPA). E) Quantification of viral genomes in the supernatant at 72 h post VPA reactivation by qPCR using primers for viral gene, ORF39, from BCBL1 treated with 10μM Brg1 inhibitor for 24 h followed by addition of 1mM VPA. F) RT-qPCR analysis of viral gene, ORF50, at 24 h post reactivation with actin as housekeeping gene (normalized to NS siRNA) in BCBL1 cells treated with 10μM Brg1 inhibitor for 24 h followed by 1mM VPA treatment (for reactivation). p values are the result of Student’s t tests and error bars indicate the standard deviation from three independent replicates.

Since Brg1 occupancy at the RTA promoter was reduced with DDX5 and DDX17 depletion, we next wanted to test whether Brg1 was required for KSHV lytic reactivation. We utilized Brg1/Brm inhibitor-1 (compound 14 or BRM014) to target Brg1 and treated iSLK.219 cells with the inhibitor either 24 h prior to reactivation or at the time of reactivation. We observed a dose-dependent reduction in viral protein expression with increasing concentration of the inhibitor in iSLK.219 cells treated with doxycycline (Fig 6A). The BRM014 inhibitor-treated iSLK.219 cells also had fewer RFP positive cells compared to the DMSO control upon lytic reactivation (Fig 6B). Next, we assessed if the infectivity of the virions produced was altered in the inhibitor treated iSLK.219 cells. We found that BRM014 inhibitor treatment of doxycycline-treated iSLK.219 cells reduced the infectivity of virions produced as measured by the supernatant transfer from iSLK.219 cells to uninfected 293 cells (Fig 6C). We also tested the inhibitor in the PEL cell line, BCBL1, and found reduced viral protein expression and fewer virions produced in the inhibitor-treated cells during lytic reactivation (Fig 6D, E). Additionally, we assessed the impact of Brg1 inhibition on RTA transcription in BCBL1 cells. Upon treatment of BCBL1 cells with the BRM014 inhibitor, we observed decreased transcription of RTA during lytic reactivation (Fig 6F). Hence, Brg1 modulates RTA transcription and is important for lytic reactivation of KSHV.

### DDX5, DDX17 and Brg1 are required for EBV lytic reactivation

**Fig 7:**
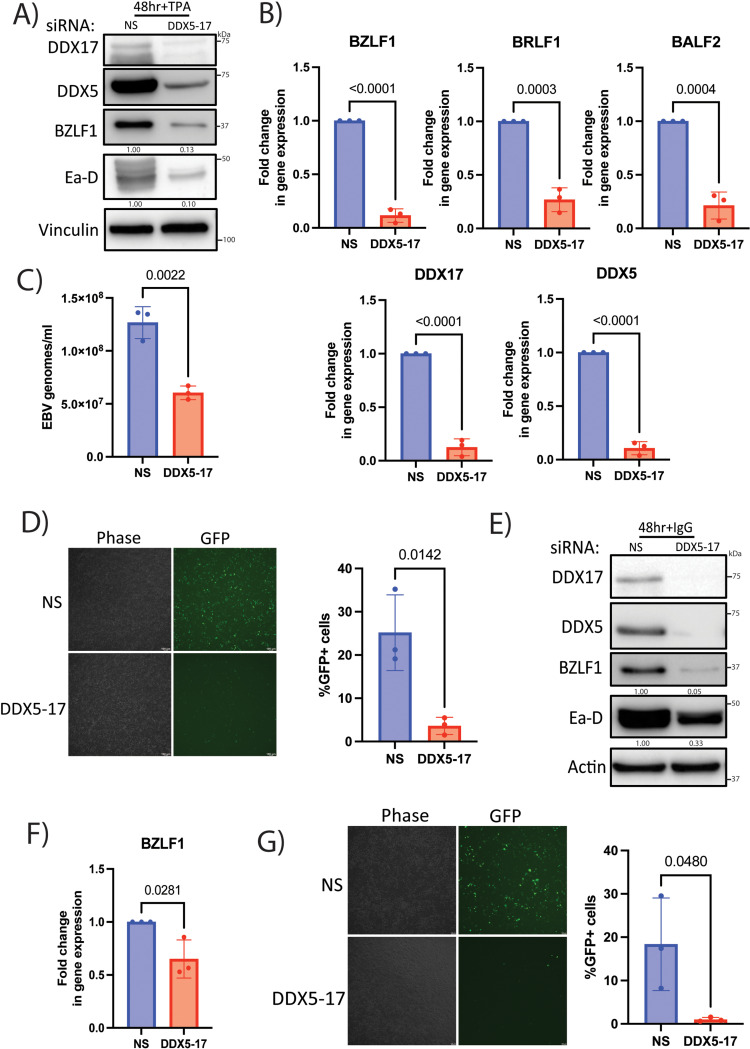
DDX5 and DDX17 are required for EBV lytic reactivation. AGS-EBV cells transfected with NS or DDX5-17 siRNAs and treated with 5ng/ml TPA 48 h post transfection. A) Western blot analysis of viral proteins BZLF1, Ea-D and host proteins DDX5, DDX17 and vinculin (loading control) at 48 h post TPA addition. B) RT-qPCR analysis of viral genes BZLF1, BRLF1 and BALF2 and host genes DDX5, DDX17 with actin as loading control (normalized to NS siRNA) at 48 h after TPA addition. C) Quantification of viral genomes in the supernatant of AGS-EBV cells transfected with NS or DDX5-17 siRNA and treated with 5ng/ml TPA. Quantification of viral genomes was performed by qPCR using primers for viral gene, BMRF1. D) Phase contrast and GFP images of 293 cells subjected to the supernatant transfer assay at 48h post transfer. Supernatant was transferred at 72 h post TPA treatment from reactivated AGS-EBV cells transfected with NS or DDX5-17 siRNAs and treated with 5ng/ml TPA as described above onto naïve, uninfected 293 cells. Quantification of virion infectivity from supernatant transfer assay by flow cytometry at 48 h post transfer. Akata-BX1 cells transfected with NS or DDX5-17 siRNAs and treated with 10μg/ml human IgG 48 h post transfection. E) Western blot analysis for viral proteins BZLF1, Ea-D and host proteins DDX5, DDX17 and actin (loading control) at 48 h post IgG addition. F) RT-qPCR analysis of viral gene BZLF1 with actin as loading control (normalized to NS siRNA) at 48 h after IgG addition. G) Phase contrast and GFP images of 293 cells subjected to the supernatant transfer assay at 48h post transfer. Supernatant was transferred at 72 h post IgG treatment from reactivated Akata-BX1 cells onto naïve, uninfected 293 cells. Quantification of virion infectivity from the supernatant transfer assay by flow cytometry at 48 h post transfer. p values are the result of Student’s t tests and error bars indicate the standard deviation from three independent replicates.

As the DDX5-17 proteins were important for KSHV lytic reactivation and also interacted with EBV BGLF4, we next tested whether the DDX5 and DDX17 proteins were required for the lytic reactivation of EBV. We transfected NS or DDX5-17 siRNA into AGS-EBV cells, an epithelial gastric carcinoma cell line latently infected with recombinant EBV expressing GFP, and reactivated EBV using 12-O-tetradecanoyl-phorbol-13-acetate (TPA). We found that there was reduced expression of early lytic proteins BZLF1 and Ea-D (BMRF1) in the DDX5-17 transfected cells compared to the NS control cells (Fig 7A). We also assessed transcription of EBV viral genes and found reduced transcription of BZLF1, BRLF1, BALF2 and BRRF1 upon DDX5 and DDX17 knockdown with lytic induction confirmed by measuring viral transcripts in latent and lytic samples (Figs 7B and S8A, B). Furthermore, there was less virion production in the supernatant and a reduced infectivity of the virions for the DDX5-17 siRNA transfected cells compared to NS siRNA control (Fig 7C, D). Next, we assessed whether lytic reactivation was also impacted when DDX5 and DDX17 were depleted in Akata-BX1 cells, a B cell line that is latently infected with GFP-expressing EBV. Similar to the AGS-EBV cell line, we saw decreased expression of early lytic viral proteins, reduced viral transcription of BZLF1, BALF2, and BRRF1, and reduced infectious virus production in the DDX5-17 depleted cells when lytic reactivation was induced using immunoglobulin (IgG) treatment (Figs 7E–G and S8C–E). Importantly, there was no difference in cell viability of AGS-EBV or Akata-BX1 cells transfected with NS or DDX5-17 siRNA (S2D, E Fig). Since we observed a decrease in transcription of the lytic EBV genes, we tested whether Brg1 is recruited to the EBV lytic promoters by conducting ChIP assays at the Z and R promoter during latency and lytic reactivation. At 18h post reactivation, we observed enrichment of Brg1 at both the Z and R promoters of EBV in the AGS-EBV cell line (S9A, B Fig).

**Fig 8:**
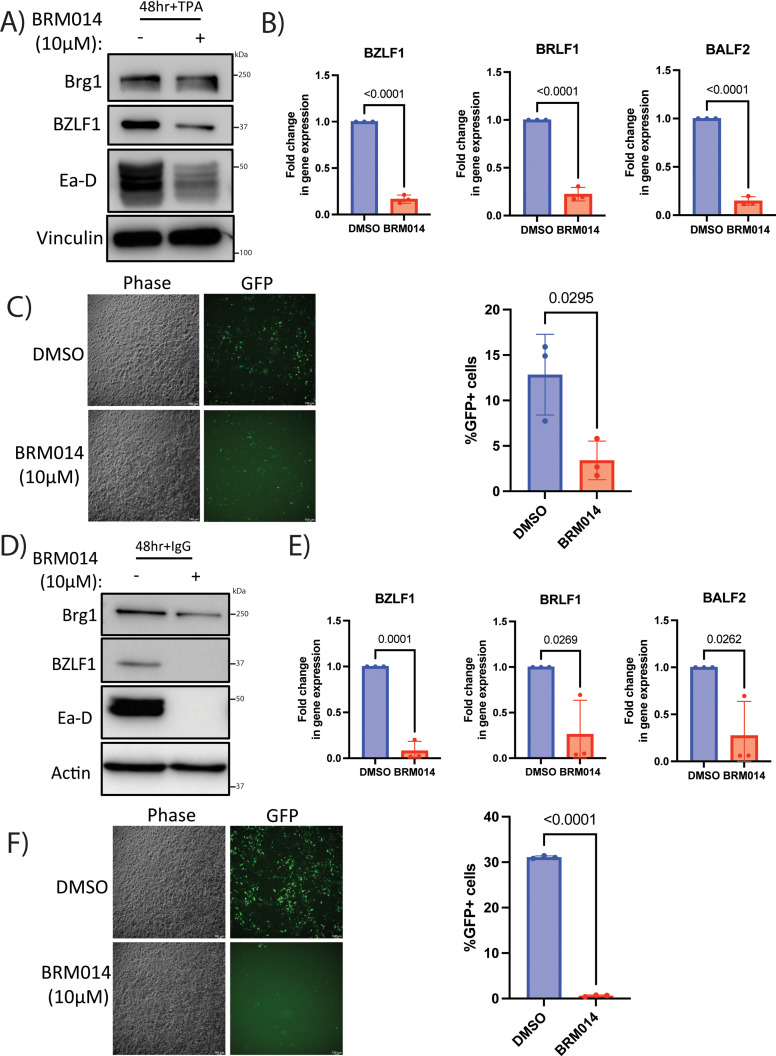
Brg1 is required for EBV lytic reactivation. AGS-EBV cells treated with 10μM BRM014 inhibitor for 24 h followed by treatment with 5ng/ml TPA. A) Western blot analysis of viral proteins BZLF1, Ea-D and host proteins Brg1 and vinculin (loading control) at 48h post TPA treatment and B) RT-qPCR analysis of viral genes BZLF1, BRLF1 and BALF2 with actin as loading control (normalized to NS siRNA) at 48 h after TPA addition. C) Phase contrast and GFP images of 293 cells subjected to the supernatant transfer assay at 48 h post transfer. Supernatant was transferred at 72 h post TPA treatment from reactivated AGS-EBV cells treated with 10μM BRM014 inhibitor and addition of 5ng/ml TPA as above onto naïve, uninfected 293 cells. Quantification of virion infectivity from the supernatant transfer assay by flow cytometry at 48 h post transfer. Akata-BX1 cells treated with 10μM BRM014 inhibitor for 24 h followed by treatment with 10μg/ml human IgG for reactivation, D) Western blot analysis of viral proteins BZLF1, Ea-D and host proteins Brg1 and actin (loading control) at 48 h post IgG addition. E) RT-qPCR analysis of viral genes BZLF1, BRLF1 and BALF2 with actin as loading control (normalized to NS siRNA) at 120 h post IgG addition. F) Phase contrast and GFP images of 293 cells subjected to the supernatant transfer assay at 48 h post transfer. Supernatant was transferred at 72 h post IgG treatment from reactivated Akata-BX1 cells treated with 10μM BRM014 inhibitor for 24 h and addition of 10μg/ml human IgG as above onto naïve, uninfected 293 cells. Quantification of virion infectivity from the supernatant transfer assay by flow cytometry at 48 h post transfer. p values are the result of Student’s t tests and error bars indicate the standard deviation from three independent replicates.

Since the DDX5 and DDX17 proteins can recruit Brg1, we next tested whether the Brg1 inhibitor would impair lytic reactivation of EBV as well. Upon addition of the BRM014 inhibitor to the AGS-EBV and Akata-BX1 cells, we observed lower expression of viral proteins BZLF1 and Ea-D, reduced viral gene transcription for BZLF1, BRLF1 and BALF2 and reduced infectious virion production in both cell lines upon reactivation (Fig 8A–F). We also depleted Brg1 using siRNA and found reduced viral protein expression for both KSHV and EBV cell lines (S9C–F Fig). Thus, EBV utilizes DDX5, DDX17 and Brg1 for optimal lytic reactivation, similar to KSHV.

## Discussion

Gammaherpesviruses cause lifelong infections in humans and are linked to epithelial, endothelial, and B cell cancers among others. The viruses establish a cellular environment conducive for latency or lytic replication by encoding for viral proteins that directly modulate or mimic the function of host proteins. These virus-host interactions aid the virus to evade host immunity and allow viral DNA replication and virion production to complete the viral lifecycle [3]. The viral proteins play important roles during latency and lytic replication and contribute to gammaherpesvirus-associated diseases. The viral protein kinases are a group of highly conserved, serine/threonine kinases encoded by all human herpesviruses [23]. For gammaherpesviruses, this includes KSHV’s viral protein kinase (vPK), encoded by ORF36, and EBV-encoded BGLF4. vPK and BGLF4 are both packaged within virions and important for the modulation of the innate immune and DNA damage response, and viral gene expression and infectious virion production [18,19,23,28,31,56]. Moreover, vPK mimics the function of host kinase S6KB1, sharing many cellular targets with S6KB1 [16]. Importantly, transgenic mice overexpressing vPK show increased incidence of lymphomas [21]. This points to the crucial role of viral protein kinases in creating an optimal environment for gammaherpesvirus infection. To fully unravel the role of vPK in the lifecycle of KSHV, we investigated the host binding partners of vPK and identified DDX5 and DDX17 as vPK’s interacting partners. We also found that the interaction of DDX5 and DDX17 was conserved for EBV’s BGLF4. Furthermore, we show that these two proteins influence lytic reactivation of KSHV and EBV. This demonstrates a conserved function of gammaherpesviral protein kinases to interact with DDX5 and DDX17 and utilize these host proteins for viral infection. While the present study is focused on gammaherpesviruses, it would be interesting to determine if other herpesviral protein homologs also interact with DDX5 and DDX17. Interestingly, DDX17 was reported to be a binding partner of HCMV’s UL97, a homolog of vPK, in a mass-spectrometry-based interactome study [57]. It is fascinating to speculate if DDX5 and DDX17 would play similar proviral roles in other herpesvirus infections.

vPK has been shown to aid in lytic replication by phosphorylation of KAP-1, a transcription repressor that places heterochromatin marks on viral promoters in the KSHV genome [22]. Additionally, vPK can bind to the ORF57 and K-bZIP promoters as well as OriLyt [26]. In our study, we show that vPK can bind to the RTA promoter and interacts with DDX5 and DDX17. We also show that DDX5 and DDX17 are present at the RTA promoter and promote Brg1 binding, a member of the chromatin remodeling complex, to the RTA promoter during lytic reactivation. Similarly, the homolog of vPK in HCMV, UL97, and *orf36* in MHV-68 can also phosphorylate histone deacetylases to allow for immediate early (IE) or RTA expression, respectively [58,59]. Importantly, these observations allude to a mechanism where vPK can modify the chromatin-remodeling proteins that bind to a viral promoter, similar to its role with KAP-1, and regulate viral gene expression. Moreover, vPK and the IE viral protein K8 interact with each other and regulate viral gene expression [26]. Interestingly, another study reported that DDX5 and DDX17 are some of the cellular proteins that interact with KSHV K8, although their role in the context of K8 function was not delineated [60]. This suggests that vPK, DDX5 and DDX17 may regulate viral gene expression as part of protein complexes including other viral genes like K8 and have a broad impact on KSHV gene expression. Future studies will examine the interplay between DDX17, DDX5, vPK and Brg1 at other viral promoters and determine how they regulate viral gene transcription. We will also assess how vPK affects cellular gene expression, which may in turn influence lytic reactivation. Thus, our study reaffirms the important role of viral protein kinases in promoting KSHV infection.

DDX/DHX proteins belong to a large family of RNA helicases that are important for RNA metabolism within the cell. DDX5 and DDX17 are two paralogous members of this family whose functions span several RNA processes such as miRNA processing, RNA decay, ribosomal RNA biogenesis and transcriptional modulation [32–34]. Both DDX5 and DDX17 proteins have been implicated in regulating RNA viral infections [47]. DDX5 and DDX17 both influence viral replication of human immunodeficiency virus (HIV), HBV and influenza [44,48,61–64]. However, these proteins can singularly affect RNA virus infections as well. DDX17 aids bunyavirus infection while DDX5 affects hepatitis C virus (HCV), SARS-CoV and Japanese encephalitis virus (JEV) replication [45–47,65]. The roles of these proteins in DNA virus infections are less studied. DDX17 has been reported to be proviral for herpes simplex virus 1 (HSV-1) infection by transcriptional regulation of early genes [66]. Here, we describe the role of the DDX5 and DDX17 proteins in augmenting gammaherpesvirus lytic reactivation. For KSHV, we found that these two proteins are localized to the nucleus during lytic reactivation and modulate transcription of RTA. Previously, DDX5 and DDX17 have been reported to be localized in the replication and transcription compartments of KSHV during viral lytic reactivation [67]. Furthermore, in HSV-1 infection, DDX17 is localized to viral transcription compartments in the nucleus of the infected cell [66]. These reports are in line with our observations of the nuclear localization of DDX5 and DDX17 during KSHV lytic reactivation, and their function during gammaherpesviral lytic reactivation.

DDX5 and DDX17 proteins are often part of large multi-subunit complexes, and they can act as transcription activators or repressors depending on their interacting partners, independent of their RNA helicase activity [32,34,40,68]. Interestingly, DDX5 and DDX17 exhibit functional redundancy for transcriptional regulation [34,40,68]. We found that DDX5 and DDX17 are functionally redundant during KSHV lytic reactivation, as has been previously described for other transcriptional modulation by DDX5 and DDX17 [40,43,68–71]. In addition, we see a transcription-enhancing role for the DDX5-17 proteins during gammaherpesviral lytic reactivation and our study adds KSHV and EBV viral genes to the list of genes regulated by DDX5 and DDX17. We also find that DDX5 and DDX17 are important for primary infection of KSHV in endothelial cells. Since there is a wave of lytic gene expression prior to establishment of latency during primary infection, the DDX5-17 proteins could be regulating RTA transcription and primary infection in a similar manner [53]. Thus, our study demonstrates the importance of DDX5 and DDX17 proteins in gammaherpesviral lytic reactivation by regulating viral gene expression.

RTA, encoded by ORF50, is the key gene to switch from latency to lytic reactivation of KSHV. RTA expression is modulated by various host factors that can regulate its transcription via assembly of epigenetic machinery and transcription regulators. The SWI/SNF (SWItch/ sucrose non-fermentable) complex is a highly conserved group of proteins that influence transcription by altering the chromatin accessibility [72]. This large complex is composed of various protein members with an ATPase subunit required for its chromatin remodeling function [72]. Brg1 or Brm proteins serve as the ATPases that move the chromatin remodelers over DNA via ATPase activity [73]. Nucleosome remodeling occurs during the latency to lytic switch and Ini1/SNF5, a member of the SWI/SNF complex, binds to the RTA promoter during lytic reactivation [13]. Interestingly, SWI/SNF complex members can influence RTA expression and lytic reactivation. Gwack et al. reported that the members of the SWI/SNF complex interacted with RTA, and that Brg1 could directly interact with RTA and regulate the transcription of RTA-dependent viral genes [55]. Also, Brg1 has been shown to be important for late viral gene expression in EBV NPC cell lines [74]. Furthermore, DDX5 and DDX17 were reported to recruit chromatin remodeling and transcription initiation machinery to cellular promoters [34]. These included RNA polymerase II (RNA pol II), CREB-binding protein (CBP), p300, Brg1 and HDACs which influence transcription of genes [40,75]. In our study, we find that DDX5 and DDX17 are important for Brg1 occupancy at the KSHV viral RTA promoter. Using the in vitro promoter binding assay, we also found that the DDX proteins and vPK, but not Brg1, can be directly recruited to viral promoters. This suggests that the SWI/SNF complex assembly at the RTA promoter might require other viral factors, such as RTA, or might bind to promoters in a chromatinized viral genome during lytic reactivation. Based on these findings, we propose a model where vPK and DDX proteins are recruited to the lytic viral promoters and promote binding of chromatin remodeling proteins, such as Brg1. Furthermore, it is possible that the loss of Brg1 upon DDX depletion may hinder optimal chromatin remodeling and further alter occupancy of transcription machinery and other proteins at viral promoters. In addition, we show that Brg1 is important for IE and early viral gene expression during lytic reactivation of gammaherpesviruses. Thus, we highlight the crucial role of SWI/SNF member Brg1 in gammaherpesviral lytic reactivation and demonstrate its importance in modulation of viral gene expression.

**Fig 9:**
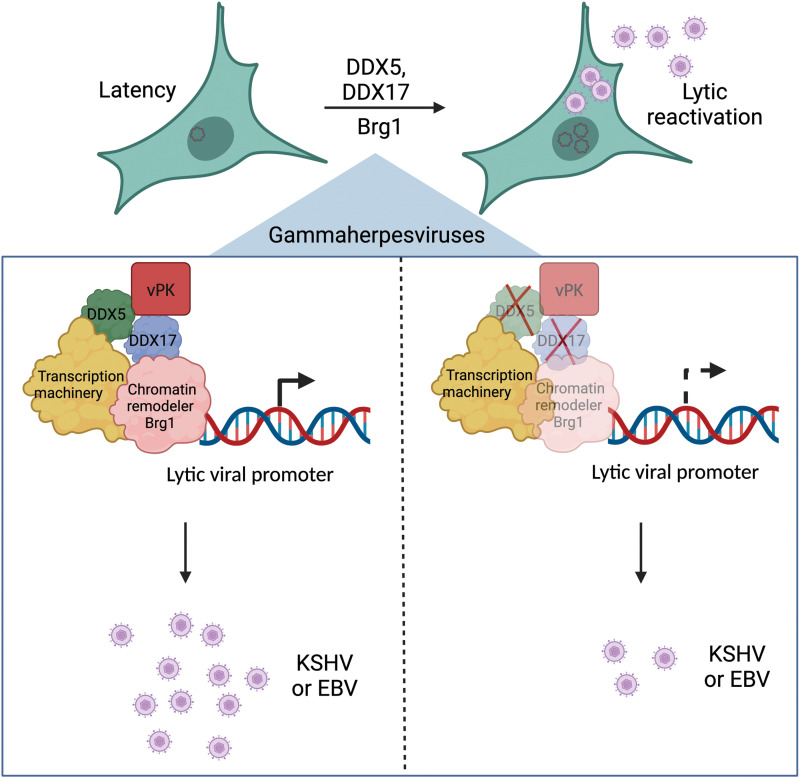
Proposed model depicting the role of vPK, DDX5, DDX17 and Brg1 during gammaherpesviral lytic reactivation. vPK, DDX5 and DDX17 are recruited to lytic viral promoters where the DDX proteins engage Brg1 to drive viral gene expression and lytic reactivation. Depletion of DDX5 and DDX17 reduces Brg1 and vPK binding to lytic viral promoters, reduces lytic viral gene expression and decreases lytic reactivation of gammaherpesviruses. Created in BioRender.

DDX5 and DDX17 are recruited to specific promoters to repress transcription during neuronal differentiation [39]. During skeletal muscle development, DDX5 and DDX17 interact with myoD, a transcription factor required for myogenesis, to recruit Brg1, TATA-binding protein (TBP) and RNA pol II and enable transcription of muscle development genes [40]. In addition, DDX5 can directly bind to p53 and alter p53-dependent p21 gene transcription to control the cell cycle [41,76]. In this study, we find DDX5 and DDX17 adopting a similar role to engage Brg1 and enable IE viral gene transcription during gammaherpesviral lytic reactivation. Interestingly, while Brg1 binding to the viral promoter was depleted upon knockdown of DDX5 and DDX17, we did not find reduced occupancy of another SWI/SNF complex member, Ini-1. This suggests that DDX5 and DDX17 specifically engage Brg1, and their loss does not lead to a general loss of SWI/SNF complex machinery at the RTA promoter. DDX5 and DDX17 regulate expression and splicing of NFAT5 and macroH2A1 histone proteins [69,70]. They also bind to β-catenin and regulate transcription of β-catenin dependent genes in colon cancer cells [68]. The transcriptional/splicing regulation allows DDX5 and DDX17 to induce cellular differentiation or commitment to a specific cell lineage [40,69,70]. Our results show that gammaherpesviruses co-opt the transcriptional modulation roles of DDX5 and DDX17 to drive the lytic reactivation program, similar to inducing a specific gene expression profile during differentiation or lineage commitment. In summary, we show that the viral protein kinases interact with DDX5 and DDX17, and these DDX proteins modulate gammaherpesviral reactivation by recruitment of Brg1 (Fig 9). These findings highlight the roles of host DDX proteins in gammaherpesviral infection and present a new avenue for therapeutic intervention for gammaherpesvirus-associated diseases.

## Methods

### Cell culture

Human embryonic kidney 293T and 293 cells were cultured in Dulbecco’s modified Eagle’s medium (DMEM from Thermo Fisher) supplemented with 1% penicillin-streptomycin, L-glutamine and 10% fetal bovine serum (VWR). iSLK.219 cells were cultured in DMEM (Thermo Fisher) supplemented with 1% penicillin-streptomycin, L-glutamine and 10% tetracycline-free fetal bovine serum (Clontech), 10μg/ml puromycin (Corning), 400μg/ml hygromycin B (Corning) and 250μg/ml G418 (Thermo Fisher). 293T.219 cells were cultured in Dulbecco’s modified Eagle’s medium (DMEM from Thermo Fisher) supplemented with 1% penicillin-streptomycin, L-glutamine, 10% fetal bovine serum (VWR) and 1μg/ml puromycin (Corning). iSLK-RTA cells were cultured in DMEM supplemented with 1% penicillin-streptomycin, L-glutamine, 10% tetracycline-free fetal bovine serum, 400μg/ml hygromycin B and 250μg/ml G418. BCBL1 cells were cultured in RPMI 1640 media (Corning) supplemented with 1% penicillin-streptomycin, L-glutamine and 10% fetal bovine serum, 0.05mM beta-mercaptoethanol and 1% sodium bicarbonate. TREx-BCBL1-RTA cells were cultured in RPMI 1640 media (Corning) supplemented with 200μg/ml hygromycin B, 1% penicillin-streptomycin, L-glutamine and tetracycline-free 10% fetal bovine serum, 0.05mM beta-mercaptoethanol and 1% sodium bicarbonate. HUVEC (immortalized with human telomerase reverse transcriptase (hTERT)) were cultured in Endothelial Cell Basal medium (PromoCell) supplemented with supplement kit 2 (except heparan sulfate and ascorbic acid), 10% fetal bovine serum, and 1% penicillin-streptomycin, L-glutamine. AGS-EBV cells were cultured in F-12 medium (Thermo Fisher) supplemented with 1% penicillin-streptomycin, L-glutamine, 10% fetal bovine serum and 500μg/ml G418. Akata-BX1 cells were cultured in RPMI 1640 supplemented with 1% penicillin-streptomycin, 1% L-glutamine, 10% fetal bovine serum, 0.05mM beta-mercaptoethanol and 500μg/ml G418. Cell cultures were maintained at 5% CO2 and 37°C in humidified incubators and cell lines were routinely screened for mycoplasma contamination.

### siRNA transfection

siRNAs targeting gene of interest (DDX5: J-003774-06, J-003774-07, DDX17: J-013450-9, J-013450-11, Brg1: L-010431) or control were purchased from Horizon Discovery and were dissolved in 1X siRNA buffer (Corning) to make 100μM stock solution. Transfection for siRNA was performed using RNAiMax (Thermo Fisher) for iSLK.219, HUVEC and AGS-EBV cells. Both siRNA and RNAiMax were diluted in OptiMEM (Thermo Fisher) and transfection was performed according to manufacturer’s protocol (forward and reverse transfection). Nucleofection was used for transfecting siRNA into BCBL1 and Akata-BX1 cell lines with Cell line Nucleofector kit V according to manufacturer’s guidelines. KSHV or EBV reactivation was performed in all cell lines at 48 h post siRNA transfection. siRNA sequences for dual targeting of DDX5 and DDX17 are listed in S1 Table.

### Virus propagation and reactivation

KSHV virus was harvested after reactivation in iSLK.219 cells. Briefly, iSLK.219 were expanded in T-175 flasks and reactivated in serum-free DMEM using 3μg/ml doxycycline and 1mM sodium butyrate. Supernatant was harvested 48 or 72 h post reactivation, filtered through a 0.45μM filter, overlaid on a 20% sucrose cushion and subjected to ultracentrifugation in SW32Ti rotor (Beckman Coulter) at 25000 rpm at 4°C for 2.5h. Pellet was dissolved in 1X PBS, aliquoted and stored at -80°C.

KSHV reactivation was performed in DMEM supplemented with L-glutamine and 10% fetal bovine serum containing 2μg/ml or 0.2μg/ml doxycycline in iSLK.219 cells, or using 3mM sodium butyrate and 25ng/ml TPA in 293T.219 cells. For BCBL1 cells, reactivation was induced by addition of 1mM valproic acid to culture media. EBV was reactivated in AGS-EBV cells with 5ng/ml TPA to culture media. In Akata BX1 cells, EBV was reactivated using B cell receptor crosslinking by addition of 10μg/ml human IgG.

### SDS PAGE and Western blot

Cell lysates were prepared in either 0.1% NP40 lysis buffer (0.1% NP40, 50mM Tris HCl pH8, 150mM NaCl, 30mM β-glycerophosphate, 50mM NaF) or RIPA buffer (1% NP40, 0.5% sodium deoxycholate, 0.1% SDS, 50mM Tris HCl pH8, 150mM NaCl, 30mM β-glycerophosphate, 50mM NaF). Lysates were quantitated using a BCA assay kit (Thermo Fisher) and equal amounts of protein lysates were loaded onto SDS PAGE gels, separated by size and transferred onto nitrocellulose membranes (Cytiva) for Western blotting. For membrane blocking and antibody dilution, 5% nonfat dry milk or bovine serum albumin (BSA) in TBS-T (Tris-buffered saline with 0.1% Tween 20) was used. Antibodies used for this study are as follows:

Tags: myc-hrp (Thermofisher, R951-25), HA-hrp (Cell Signaling Technology, 2999), FLAG-hrp (Sigma Aldrich, A8592), V5-hrp (Thermofisher, R961-25).

Human: DDX17 (Fortis Life Sciences, A300-509A), DDX5 (Fortis Life Sciences, A300-523A; Cell Signaling Technology, 4387S), Brg1 (Santa Cruz Biotech, G7 sc17796X), actin-hrp (Santa Cruz Biotech, sc47778).

KSHV: ORF45 (Thermofisher, MA5-14769), vIL6 (synthesized), K8α (Santa Cruz Biotech, sc57889), LANA (Advanced Biotechnologies, 13-210-100), vPK (synthesized).

EBV: BZLF1 (Santa Cruz Biotech, sc53904), Ea-D (Santa Cruz Biotech, sc69679).

### RT-qPCR

RNA was isolated from samples using the RNeasy Plus Mini RNA isolation kit (Qiagen) and equivalent amounts of RNA were used to prepare complementary DNA (cDNA) using the iScript gDNA Clear cDNA synthesis kit. cDNA was diluted for the qPCR reaction and used with the SensiFAST SYBR Lo-ROX kit (Bioline). Ct values were normalized to actin and fold change was analyzed between control and experimental samples using the ΔΔCt method. Primers used for RT-qPCR are listed in S1 Table.

### Viral supernatant transfer

Supernatants were harvested from re-activated cells at 72 h post reactivation and diluted in serum-free DMEM supplemented with L-glutamine and 10μg/ml polybrene. The diluted supernatants were added onto HEK293 cells plated in 6 well plates and spinfected at 2500rpm at 30°C for 90 minutes. Post spin, the cultures were supplemented with 1ml of DMEM supplemented with 1% penicillin-streptomycin, L-glutamine and 10% fetal bovine serum and incubated at 37°C overnight. The following day, media was changed to DMEM supplemented with 1% penicillin-streptomycin, L-glutamine and 10% fetal bovine serum and the cells were incubated at 37°C for 48 h. HEK293 cells were imaged at 48h post supernatant transfer, trypsinized, resuspended in 1X PBS and analyzed by flow cytometer MACS Quant VYB to count GFP positive cells.

### Plasmids and inhibitor

Plasmids for myc-DDX17 (Addgene 19876), Brg1-FLAG (Addgene 19143) and mThy1.1-hDDX5 (Addgene 88873) were purchased. DDX5 was cloned into pDEST with DDX17 digested out and myc tag fused to DDX5. Site directed mutagenesis was performed to switch myc to HA to generate HA-DDX5 plasmid. V5-vPK was previously generated in the lab [16]. BGLF4-3XFLAG was synthesized from VectorBuilder. BRM014 was purchased from MedChem Express (HY-119374) and re-suspended in DMSO.

### Plasmid transfection and lysate preparation

Plasmids were transfected into HEK293T cells seeded in 10 cm dishes using Xtreme Gene 9 (Roche). Transfections were carried out using 3:1 transfection reagent:DNA ratio with a total 10μg of DNA per 10 cm dish. Cells were incubated for 48 or 72 h post transfection and lysed in 1ml of RIPA buffer per 10 cm after washing with 1X PBS. Cells were lysed on ice for 20 minutes followed by centrifugation at 13000rpm for 10 minutes at 4°C. Lysates were quantitated and used for co-immunoprecipitation.

### Co-immunoprecipitation

HEK293T cells were seeded in 10 cm dishes and transfected with V5-tagged vPK, HA-tagged DDX5, myc-tagged DDX17 or BGLF4-3XFLAG plasmids. Cells were harvested at 48 or 72 h post transfection in RIPA buffer. Lysates were precleared using Protein A/G beads and control rabbit or mouse IgG. For the co-immunoprecipitation, beads (50μl) were washed and incubated with 1mg of lysate overnight. For magnetic beads, the beads were placed onto a magnetic stand for 2 minutes to separate the beads and the solution was aspirated. For agarose beads, beads were spun at 2600rpm for 5 minutes at 4°C and the supernatant was aspirated. Beads were washed four times with RIPA buffer and eluted with V5, myc, FLAG and HA peptide by incubating for 45 mins at 4°C followed by spinning or magnetic separation to remove the eluate. Next, the beads were subjected to Laemmli elution by addition of 2X Laemmli buffer followed by heating at 95°C for 5 mins. Eluates were subjected to SDS-PAGE and Western blotting to detect V5-vPK, endogenous DDX5 or DDX17, myc-DDX17, HA-DDX5 and BGLF4-3XFLAG.

### Chromatin immunoprecipitation

ChIP beads (40μl) were washed thrice with RIPA buffer and blocked with 5mg/ml bovine serum albumin (BSA) for 1 h at room temperature, followed by addition of antibody (2-4μg) and overnight incubation at 4°C. iSLK.219 or 293T.219 cells were harvested from 10 cm dishes by trypsinization without reactivation or at 24hr post doxycycline (2μg/ml) or sodium butyrate and TPA treatment, respectively. AGS-EBV cells were re-activated using 5ng/ml TPA and harvested from 10 cm dishes without reactivation or at 18hr post TPA treatment by trypsinization. The cells were washed three times in 1X PBS and fixed with 1% paraformaldehyde (Thermo Fisher) for 10 minutes at room temperature. Fixation was quenched using 125mM glycine for 5 mins at room temperature, followed by three washes with cold 1X PBS and centrifugation to pellet fixed cells. Pellets were re-suspended in lysis buffer (5mM Tris-HCl, pH 8.0, 85mM KCl, 0.5% NP-40, 1x cOmplete Protease Inhibitor (Roche) followed by incubation at 4°C for 30 minutes. Lysate was subjected to centrifugation at 3000rpm for 5 mins at 4°C, followed by aspiration of supernatant and resuspension of nuclei pellets in RIPA buffer (150mM NaCl, 1.0% NP-40, 0.5% sodium deoxycholate, 0.1% SDS, 50mM Tris-HCl, pH 8.0, 1x cOmplete Protease Inhibitor) and incubation at 4°C for 30 minutes. The chromatin from lysed nuclei was then sheared using the sonicator Diagenode Bioruptor Plus 4°C on Hi setting with 30 seconds on, 30 seconds off program using appropriate cycles for optimal shearing. The sheared chromatin was spun at 13000rpm for 10mins at 4°C and the supernatant was removed with aliquots saved for input. The supernatant was pre-cleared using Protein A/G agarose beads and then diluted in RIPA buffer. Antibody-coated and blocked ChIP beads were washed once in RIPA and incubated with chromatin for ChIP overnight at 4°C. The following day, beads were spun down to remove unbound chromatin and subjected to 2 washes each with the following buffers: RIPA, Low Salt Buffer (0.1% SDS, 1% NP-40, 2mM EDTA, 20mM Tris-HCl, pH 8.0, 150mM NaCl), High Salt Buffer (0.1% SDS, 1% NP-40, 2mM EDTA, 20mM Tris-HCl pH 8.0, 500mM NaCl), LiCl Buffer (0.25M LiCl, 1% NP-40, 1% sodium deoxycholate, 1mM EDTA, 20mM Tris-HCl, pH 8.0), and Tris-EDTA (pH8.0). Elution Buffer (100mM NaHCO3, 1% SDS) was used to elute DNA for 30min at 65˚C, followed by crosslinking reversal with de-crosslinking Buffer (500mM NaCl, 2mM EDTA, 20mM Tris-HCl, pH 8.0, 0.5mg/mL Proteinase K) overnight at 65˚C. The immunoprecipitated DNA was purified with Qiagen PCR Purification Kit following the manufacturer’s protocol. The qPCR primers used are listed in S1 Table. The antibodies used for ChIP experiments are as follows:

DDX17 (Proteintech, 19910-1-AP), DDX5 (Abcam, ab126730), Brg1 (Santa Cruz Biotech, G7 sc17796X), Ini-1/SNF5 (Santa Cruz Biotech, sc166165X), RNA polymerase II (Active Motif, 39097), V5 (Abcam 15828) and Histone H3 (Abcam, ab1791).

### Fluorescent microscopy

iSLK.219 cells were imaged for GFP and RFP at 0 h, 24 h and 48 h post doxycycline treatment using the Leica Dmi8 inverted microscope and the fluorescent intensities were calculated using the Leica analysis software at 48h. Using the fluorescence intensities, the RFP/GFP ratio was calculated. For HEK293 cells, GFP and phase images were acquired at 48 h post supernatant transfer.

iSLK.219 and TREx-BCBL1-RTA cells were cultured on poly-L-lysine coated coverslips and reactivated using doxycycline. The cells were washed thrice with 1X PBS and fixed with 4% paraformaldehyde for 15 minutes at room temperature. Fixative was removed followed by three washes with 1X PBS. Cells were permeabilized and blocked using blocking buffer (5% goat serum, 0.5% Tween20, 5% glycine in 1X PBS) with 0.3% Triton X-100 for 1 h at room temperature. Primary antibody was diluted in blocking buffer and added onto coverslips for 1 h at room temperature. Coverslips were washed thrice with 1X TBS-T for 5 minutes each. Secondary antibody was diluted in blocking buffer and added onto coverslips for 1 h at room temperature, followed by three washes with 1X TBS-T. Cells were rinsed with water once to remove salts and mounted on slides with ProLong Glass antifade with NucBlue. Cells were imaged on the Leica Dmi8 inverted microscope and an Olympus Fluoview 1000 confocal microscope. Images were processed using the Leica LAS X and Imaris software.

Primary antibodies used are DDX17 (Abcam, ab180190), DDX5 (Abcam, ab126730) and ORF59 (Advanced Biotechnologies, 13-211-100). Secondary antibodies used are goat anti-mouse IgG and goat anti-rabbit antibodies conjugated to fluorophores Alexa Fluor 647 or 586 (Thermo Fisher).

### DNase resistant viral genome assay

Supernatant from reactivated iSLK.219, BCBL1 and AGS-EBV cells was centrifuged to remove cells and treated with TURBO DNase (100U/ml supernatant) and 1X DNase buffer for 1 hour at 37°C followed by DNase inactivation by addition of EDTA and heating at 70°C for 15 minutes. The DNase-treated supernatant was used to extract DNA using the DNeasy blood and tissue kit (Qiagen) according to manufacturer’s protocol. Viral genome copy numbers were quantified by generating a standard curve by amplification of ORF39 for KSHV or BMRF1 for EBV by qPCR. For the standard curves, we used plasmid pCDNA4/TO-ORF39 (a kind gift from Dr. Britt Glaunsinger) for KSHV or pSG5-BMRF1 (Addgene 72631) for EBV. The qPCR reactions were set up with the Sensifast Lo-Rox SYBR mix (Bioline). Primer sequences used are described in S1 Table.

### KSHV primary infection

HUVEC were infected with KSHV isolated from iSLK.219 cells and equivalent amount of virus was added to HUVEC transfected with NS or DDX5-17 siRNA for 48 h. Cells were imaged from 24-72 h post infection using the Leica Dmi8 inverted microscope. At 4 h post infection, cells were harvested and DNA extraction was performed. At 72 h post infection, infected HUVEC were resuspended in 1X PBS and run on flow cytometer MACS Quant VYB to count GFP positive cells. The remaining cells were harvested and processed as lysates for WB analysis.

### DNA pulldown assay

KSHV RTA and ORF57 promoters were amplified using PCR from DNA extracted from reactivated iSLK.219 cells at 48 h with the 5’ ends of the primers labeled with biotin. The size of PCR product for the promoter was confirmed using agarose gel electrophoresis. The amplified promoters were then cleaned up using the QIAquick PCR purification kit (Qiagen). V5 tagged vPK was overexpressed in 293T cells and protein lysates were prepared from these cells at 72 h post transfection. For immunoprecipitation, kilobaseBINDER Dynabeads (Invitrogen) were prepared as per manufacturer’s guidelines. Biotinylated RTA or ORF57 promoter DNA was incubated with beads for 3h at room temperature followed by addition of V5-tagged vPK, myc-DDX17, HA-DDX5 or Brg1-FLAG lysate to the beads and biotinylated DNA mixture for overnight incubation. The beads were extensively washed with RIPA buffer the next day and elution was performed using 2X Laemmli sample buffer. The eluates were loaded onto SDS PAGE gel for electrophoresis followed by Western blotting for V5, myc, HA, FLAG, actin and vinculin.

### Statistical analysis

Statistical analysis was performed using Prism 10 software. Experiments were conducted at least three times (unless noted otherwise) and error bars depict the standard deviation from three experiments. Student’s t-test was used statistical analysis and significance was assigned for p values <0.05.

## Supporting information

S1 Fig DDX5 and DDX17 interact with KSHV vPK. Western blot showing immunoprecipitation of A) myc-DDX17 and V5-vPK using anti-V5 or anti-myc agarose beads from transfected 293T cell lysates harvested at 72 h post transfection, B) myc-DDX5 and V5-vPK using anti-V5 or anti-myc agarose beads from transfected 293T cell lysates harvested at 72 h post transfection and C) endogenous DDX17, endogenous DDX5 and V5-vPK using anti-V5 agarose or anti-mouse conjugated to beads. Immunoprecipitations were eluted using Laemmli sample loading buffer and competitive peptide as indicated. Input shows actin or vinculin as loading control (A-B n=3, C n=1).
(TIF)

S2 Fig DDX5 and DDX17 depletion does not affect doxycycline-dependent gene expression or cell viability in KSHV or EBV-infected cell lines. A) RT-qPCR analysis of viral gene RTA and host genes DDX5, DDX17 with actin as loading control (normalized to NS siRNA) in iSLK-RTA cells transfected with NS or DDX5-17 siRNA and treated with doxycycline (2μg/ml) 48 h post transfection at 0 h, 24 h and 48 h after doxycycline addition. B) Live cell counts for iSLK.219 cells transfected with NS or DDX5-17 siRNA at 48 h post transfection. C) Live cell counts for BCBL1 cells transfected with NS or DDX5-17 siRNA at 48 h post transfection. D) Live cell counts for AGS-EBV cells transfected with NS or DDX5-17 siRNA at 48 h post transfection. E) Live cell counts for Akata-BX1 cells transfected with NS or DDX5-17 siRNA at 48 h post transfection. p values are the result of Student’s t tests and error bars indicate the standard deviation from three independent replicates.
(TIF)

S3 Fig DDX5 and DDX17 decrease viral gene expression during KSHV lytic reactivation in iSLK.219 cells. iSLK.219 cells transfected with NS or DDX5-17 siRNA and treated with doxycycline (2μg/ml) 48 h post transfection. RT-qPCR analysis of viral genes PAN, ORF16, vPK, K8.1, ORF22, ORF52 and ORF42 with actin as loading control A) normalized to NS siRNA at 24 h or 48h after doxycycline addition and B) delta Ct for all viral genes without doxycycline or with doxycycline (24h or 48h). p values are the result of Student’s t tests and error bars indicate the standard deviation from three independent replicates.
(TIF)

S4 Fig DDX5 and DDX17 depletion does not alter ribosome biosynthesis in iSLK.219 cells. Schematic showing the ribosomal RNA transcripts that encode for ribosomal subunits. The 47S transcript gives rise to 18S, 28S and 5.8S by splicing while the 5S is transcribed separately. RT-qPCR analysis of host genes 47S, 5.8S, 28S, 18S, 5S, DDX5, DDX17 with actin as loading control (normalized to NS siRNA) in iSLK.219 cells transfected with NS or DDX5-17 siRNA and treated with doxycycline (2μg/ml) 48 h post transfection at 0 h and 24 h after doxycycline addition. p values are the result of Student’s t tests and error bars indicate the standard deviation from three independent replicates.
(TIF)

S5 Fig DDX5 and DDX17 decrease viral gene expression during KSHV lytic reactivation in BCBL1 cells and do not affect KSHV entry in HUVEC. BCBL1 cells transfected with NS or DDX5-17 siRNA and treated with VPA (1mM) 48 h post transfection. RT-qPCR analysis of viral genes PAN, ORF16, vPK, K8.1, ORF22, ORF52 and ORF42 with actin as loading control A) normalized to NS siRNA at 24 h or 48h after VPA addition and B) delta Ct for all viral genes without VPA or with VPA (24h or 48h). C) qPCR for viral genomes at 4 h post infection from HUVEC treated with NS or DDX5-17 siRNA and infected with KSHV. Viral genomes were quantified by qPCR using primers for the viral gene, ORF39, and actin was used as a loading control. p values are the result of Student’s t tests and error bars indicate the standard deviation from two or three independent replicates.
(TIF)

S6 Fig DDX5 and DDX17 localize to the nucleus in iSLK.219 and TREx-BCBL1-RTA cells upon lytic reactivation. Fluorescent images of A) DAPI, GFP, RFP and DDX17 in iSLK.219 cells, B) DAPI, GFP, RFP and DDX5 in iSLK.219 cells and C) DAPI, ORF59, DDX17 and DDX5 in TREx-BCBL1-RTA cells at 0 h, 24 h, 48 h after doxycycline addition with higher resolution images at 24h post doxycycline in D).
(TIF)

S7 Fig vPK, DDX5, DDX17 and Brg1 bind to the RTA promoter. ChIP assay for DDX5, DDX17, Brg1, H3 or control rabbit or mouse IgG in A) latent iSLK.219 cells followed by qPCR with primers targeting 4 regions within the viral RTA promoter and a primer set targeting a gene desert in the human genome as negative control, B) reactivated iSLK.219 cells (24h post doxycycline) followed by qPCR with primers targeting 2 regions of the ORF57 promoter, C) ChIP for V5 or control rabbit IgG followed by qPCR for RTA and ORF57 promoters in 293T.219 cells at 24h post reactivation. D) Western blot for FLAG, myc, HA and V5 after DNA pulldown assay using biotinylated RTA and ORF57 promoters incubated with Brg1-FLAG, myc-DDX17, HA-DDX5 and V5-vPK lysate in the presence of poly dI:dC (10μg/ml). Input blot depicts expression of proteins using actin as a loading control. E) ChIP assay for V5 or control rabbit antibody followed by qPCR for RTA and ORF57 promoters using 293T.219 cells transfected with NS or DDX5-17 siRNAs and overexpressing V5-vPK at 24 h post reactivation, normalized to NS.
(TIF)

S8 Fig DDX5 and DDX17 are required for EBV lytic reactivation. RT-qPCR analysis showing A) Delta Ct of all viral genes in NS or DDX5-17 transfected AGS-EBV cells, B) Fold change of viral gene BRRF1 in NS or DDX5-17 siRNA transfected AGS-EBV cells at 48 h post TPA treatment, C) Fold change of cellular genes DDX5, DDX17 in NS or DDX5-17 siRNA transfected Akata-BX1 cells at 48 h post IgG treatment, D) Fold change of viral genes BRLF1, BALF2 and BRRF1 in NS or DDX5-17 siRNA transfected Akata-BX1 cells at 48 h post IgG treatment with actin as loading control (normalized to NS siRNA) and E) Delta Ct of all viral genes in NS or DDX5-17 transfected Akata-BX1 cells. p values are the result of Student’s t tests and error bars indicate the standard deviation from three independent replicates.
(TIF)

S9 Fig DDX5 and DDX17 recruit Brg1 to the RTA and ZTA promoters, and Brg1 is important for gammaherpesviral lytic reactivation. ChIP assay in A) latent AGS-EBV cells (n=1) or B) reactivated AGS-EBV cells (18h post 5ng/ml TPA treatment, n=3) using Brg1 or control mouse antibody and qPCR with primers for 4 different regions for the viral RTA (R) and 3 different regions for the viral ZTA (Z) promoter. Amplification of the gene desert is used as a control. Western blot analysis showing viral protein expression in NS or Brg1 siRNA transfected cells using C) iSLK.219 cells for ORF45, K8a, vIL6 at 24h post doxycycline treatment, D) BCBL1 cells for ORF45, K8a, vIL6 at 24h post VPA treatment, E) AGS-EBV cells for BZLF1 and Ea-D at 48h post TPA treatment and F) Akata BX1 cells for BZLF1 and Ea-D at 48h post IgG treatment. p values are the result of Student’s t tests and error bars indicate the standard deviation from three independent replicates.
(TIF)

S1 Table Primer and siRNA sequences. (DOCX)

S1 Data Source data. (XLSX)

S2 Data Uncropped blots. (PDF)
