# Supplementary figures and images for: RNA helicases, DDX5 and DDX17, facilitate lytic reactivation of gammaherpesviruses
Source: PLoS Pathog. 2025 Apr 21;21(4):e1013009. doi: 10.1371/journal.ppat.1013009 (PMC12011273; doi:10.1371/journal.ppat.1013009)

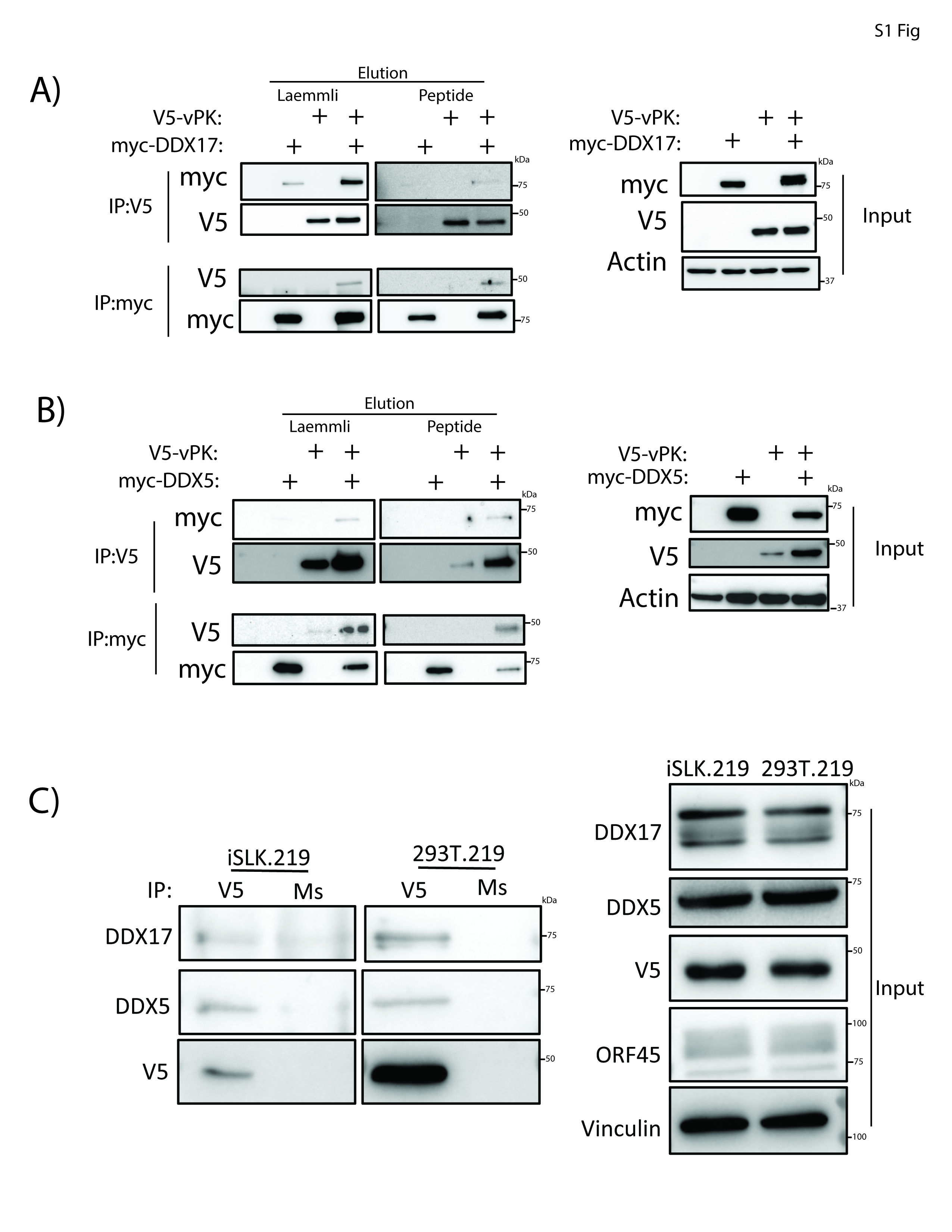

Supplement: S1 Fig — Western blot showing immunoprecipitation of A) myc-DDX17 and V5-vPK using anti-V5 or anti-myc agarose beads from transfected 293T cell lysates harvested at 72 h post transfection, B) myc-DDX5 and V5-vPK using anti-V5 or anti-myc agarose beads from transfected 293T cell lysates harvested at 72 h post transfection and C) endogenous DDX17, endogenous DDX5 and V5-vPK using anti-V5 agarose or anti-mouse conjugated to beads. Immunoprecipitations were eluted using Laemmli sample loading buffer and competitive peptide as indicated. Input shows actin or vinculin as loading control (A-B n=3, C n=1). (TIF) [file ppat.1013009.s001.tif]

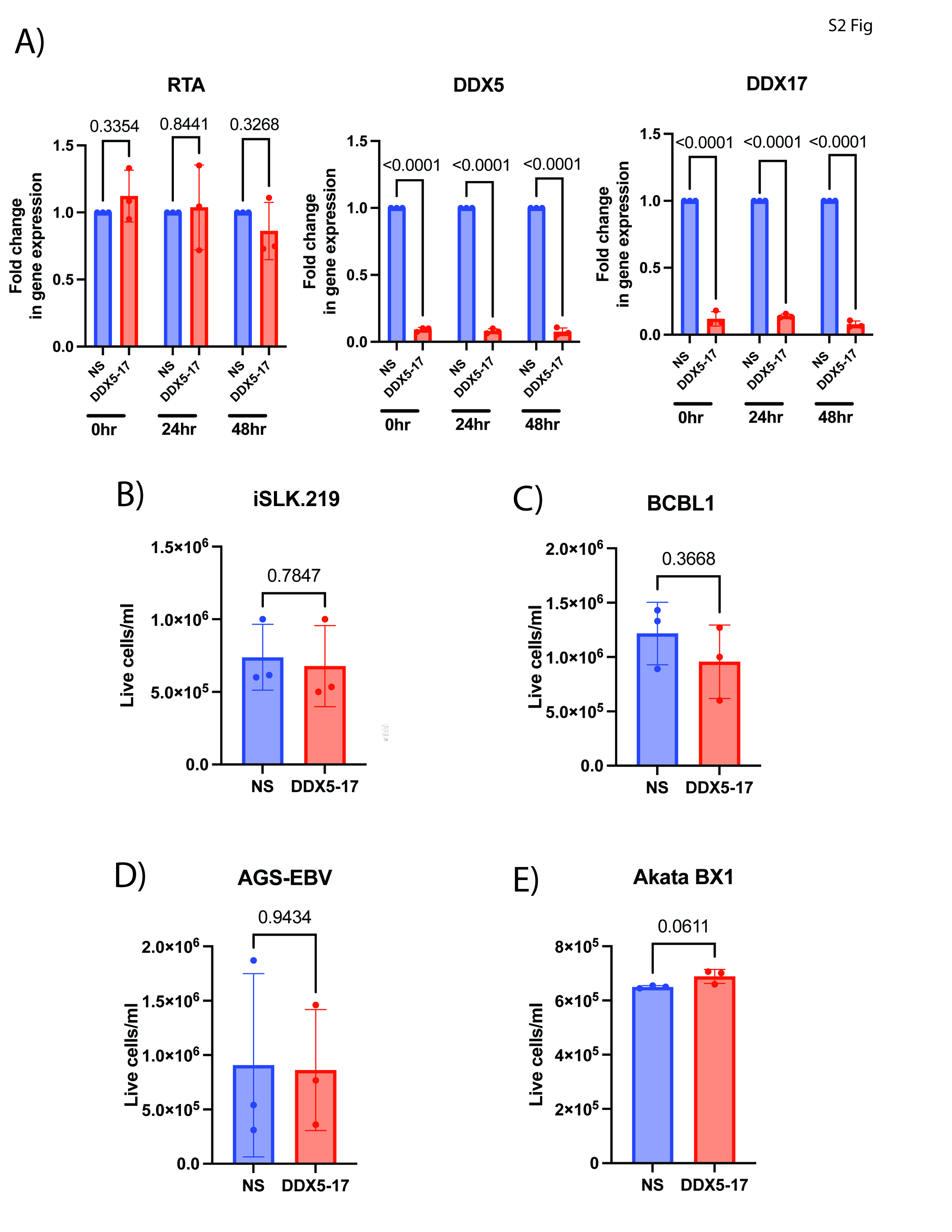

Supplement: S2 Fig — A) RT-qPCR analysis of viral gene RTA and host genes DDX5, DDX17 with actin as loading control (normalized to NS siRNA) in iSLK-RTA cells transfected with NS or DDX5-17 siRNA and treated with doxycycline (2μg/ml) 48 h post transfection at 0 h, 24 h and 48 h after doxycycline addition. B) Live cell counts for iSLK.219 cells transfected with NS or DDX5-17 siRNA at 48 h post transfection. C) Live cell counts for BCBL1 cells transfected with NS or DDX5-17 siRNA at 48 h post transfection. D) Live cell counts for AGS-EBV cells transfected with NS or DDX5-17 siRNA at 48 h post transfection. E) Live cell counts for Akata-BX1 cells transfected with NS or DDX5-17 siRNA at 48 h post transfection. p values are the result of Student’s t tests and error bars indicate the standard deviation from three independent replicates. (TIF) [file ppat.1013009.s002.tif]

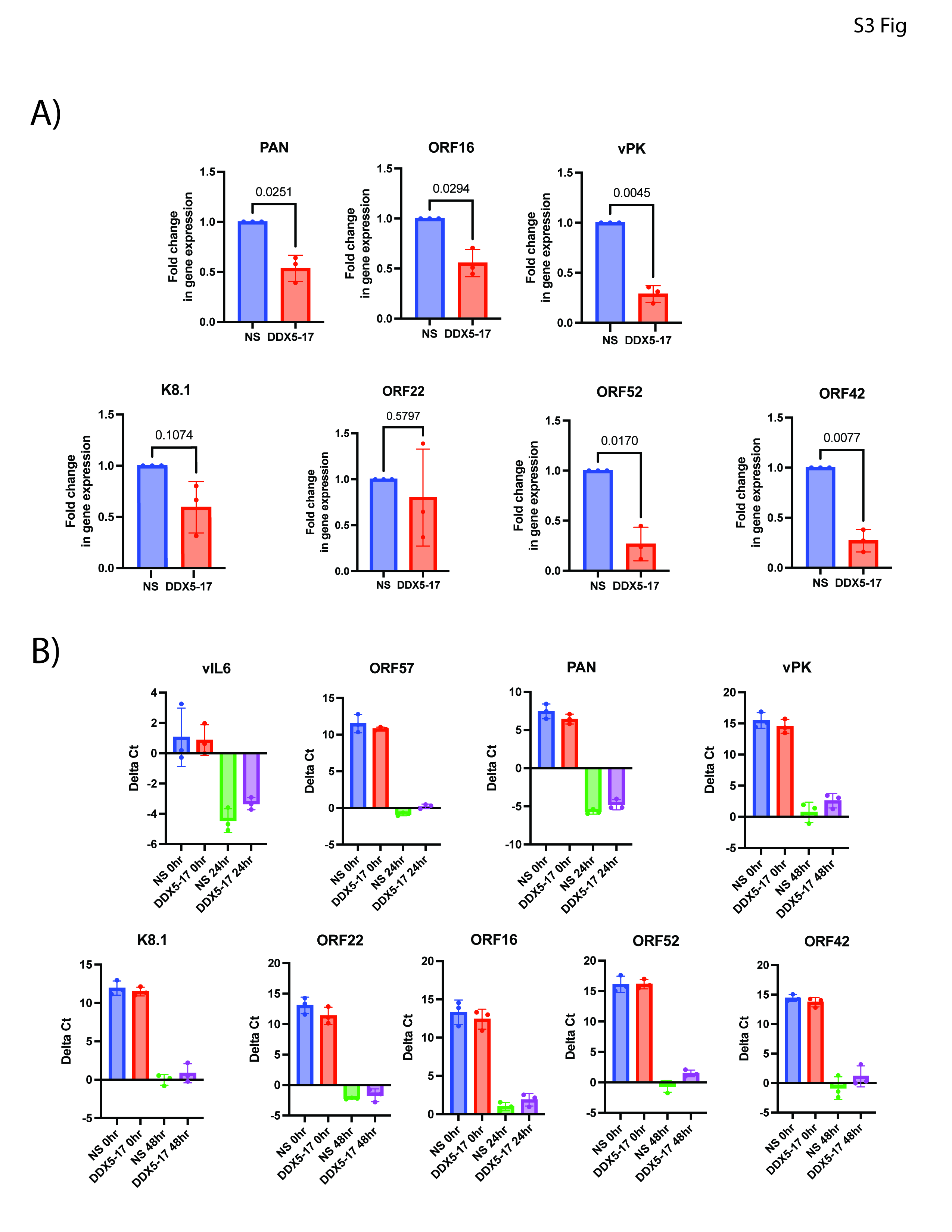

Supplement: S3 Fig — iSLK.219 cells transfected with NS or DDX5-17 siRNA and treated with doxycycline (2μg/ml) 48 h post transfection. RT-qPCR analysis of viral genes PAN, ORF16, vPK, K8.1, ORF22, ORF52 and ORF42 with actin as loading control A) normalized to NS siRNA at 24 h or 48h after doxycycline addition and B) delta Ct for all viral genes without doxycycline or with doxycycline (24h or 48h). p values are the result of Student’s t tests and error bars indicate the standard deviation from three independent replicates. (TIF) [file ppat.1013009.s003.tif]

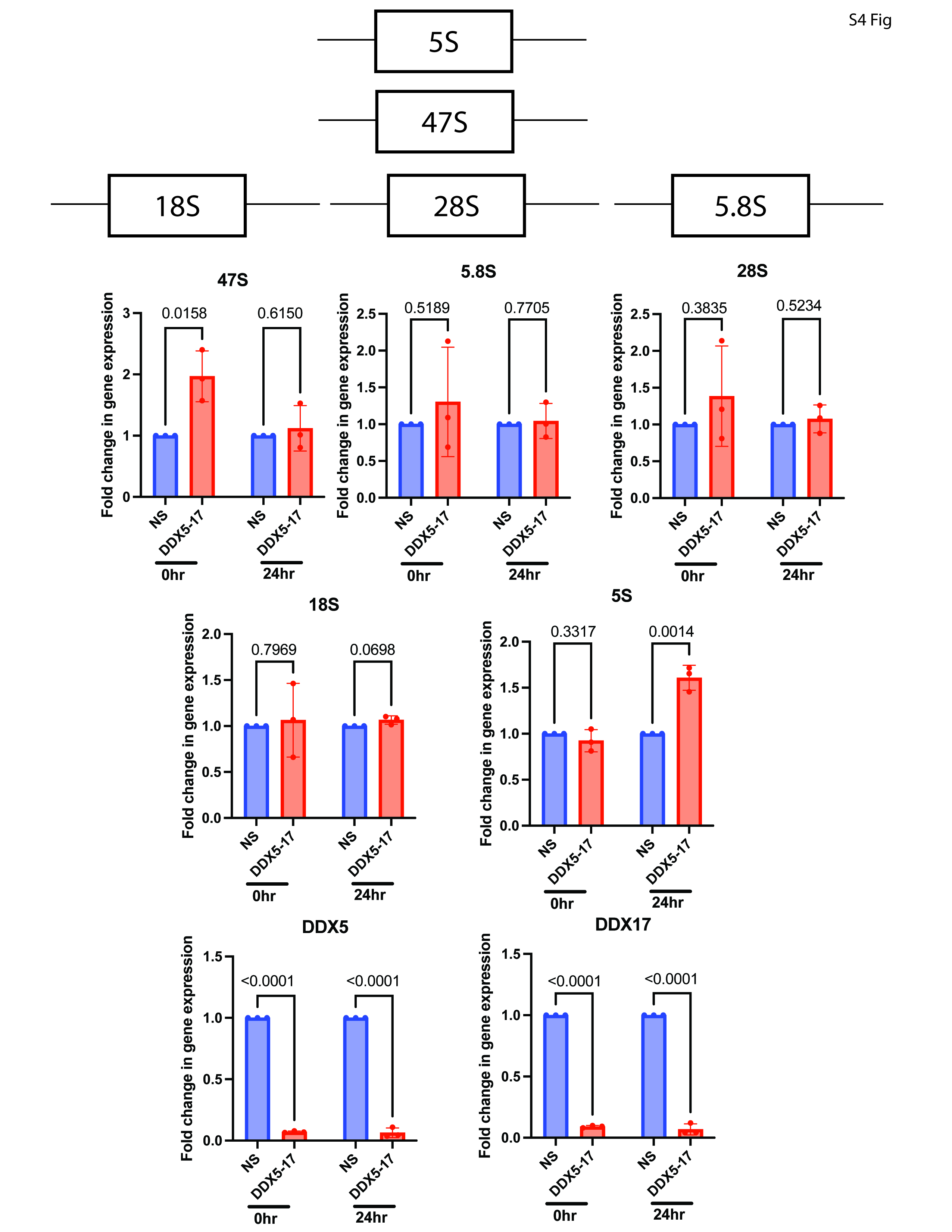

Supplement: S4 Fig — Schematic showing the ribosomal RNA transcripts that encode for ribosomal subunits. The 47S transcript gives rise to 18S, 28S and 5.8S by splicing while the 5S is transcribed separately. RT-qPCR analysis of host genes 47S, 5.8S, 28S, 18S, 5S, DDX5, DDX17 with actin as loading control (normalized to NS siRNA) in iSLK.219 cells transfected with NS or DDX5-17 siRNA and treated with doxycycline (2μg/ml) 48 h post transfection at 0 h and 24 h after doxycycline addition. p values are the result of Student’s t tests and error bars indicate the standard deviation from three independent replicates. (TIF) [file ppat.1013009.s004.tif]

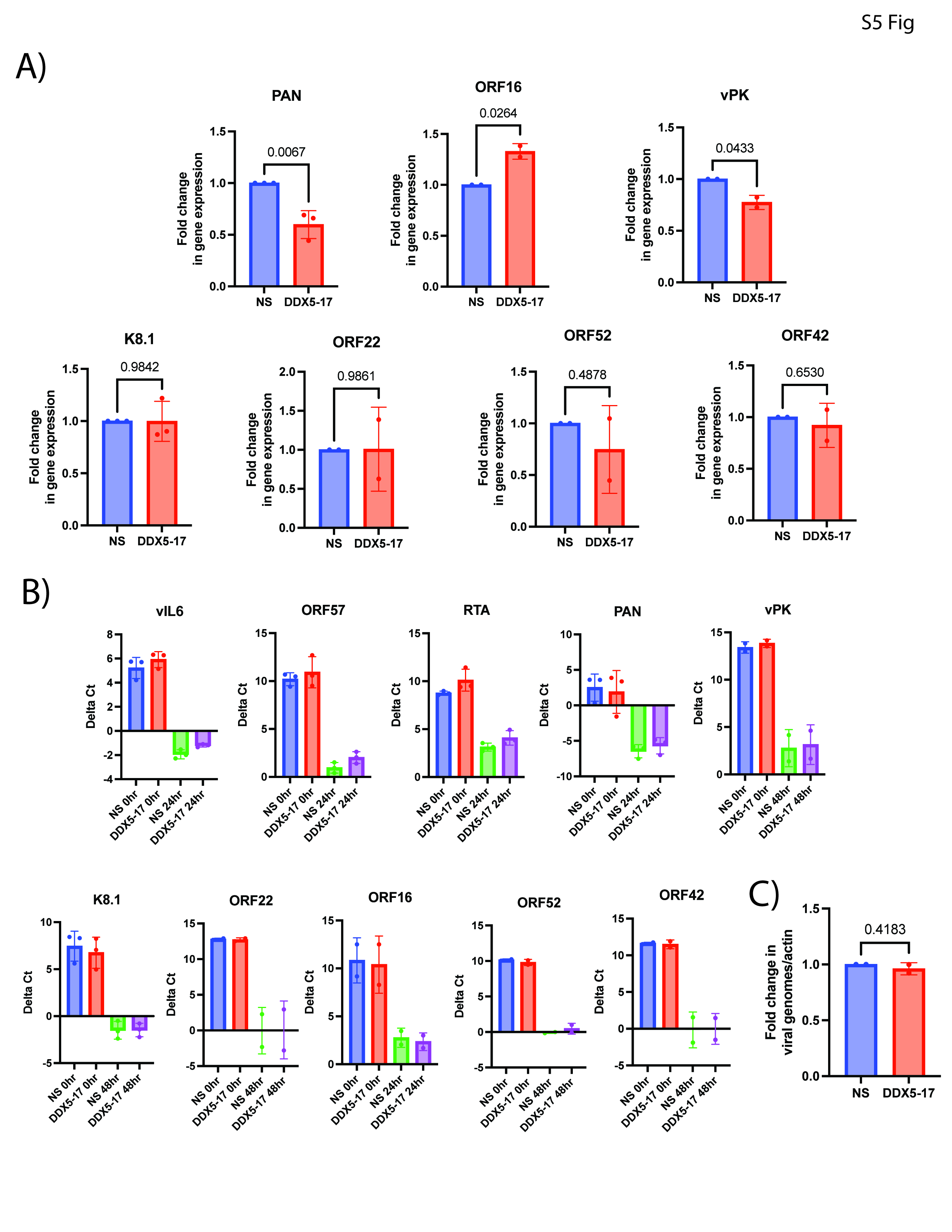

Supplement: S5 Fig — BCBL1 cells transfected with NS or DDX5-17 siRNA and treated with VPA (1mM) 48 h post transfection. RT-qPCR analysis of viral genes PAN, ORF16, vPK, K8.1, ORF22, ORF52 and ORF42 with actin as loading control A) normalized to NS siRNA at 24 h or 48h after VPA addition and B) delta Ct for all viral genes without VPA or with VPA (24h or 48h). C) qPCR for viral genomes at 4 h post infection from HUVEC treated with NS or DDX5-17 siRNA and infected with KSHV. Viral genomes were quantified by qPCR using primers for the viral gene, ORF39, and actin was used as a loading control. p values are the result of Student’s t tests and error bars indicate the standard deviation from two or three independent replicates. (TIF) [file ppat.1013009.s005.tif]

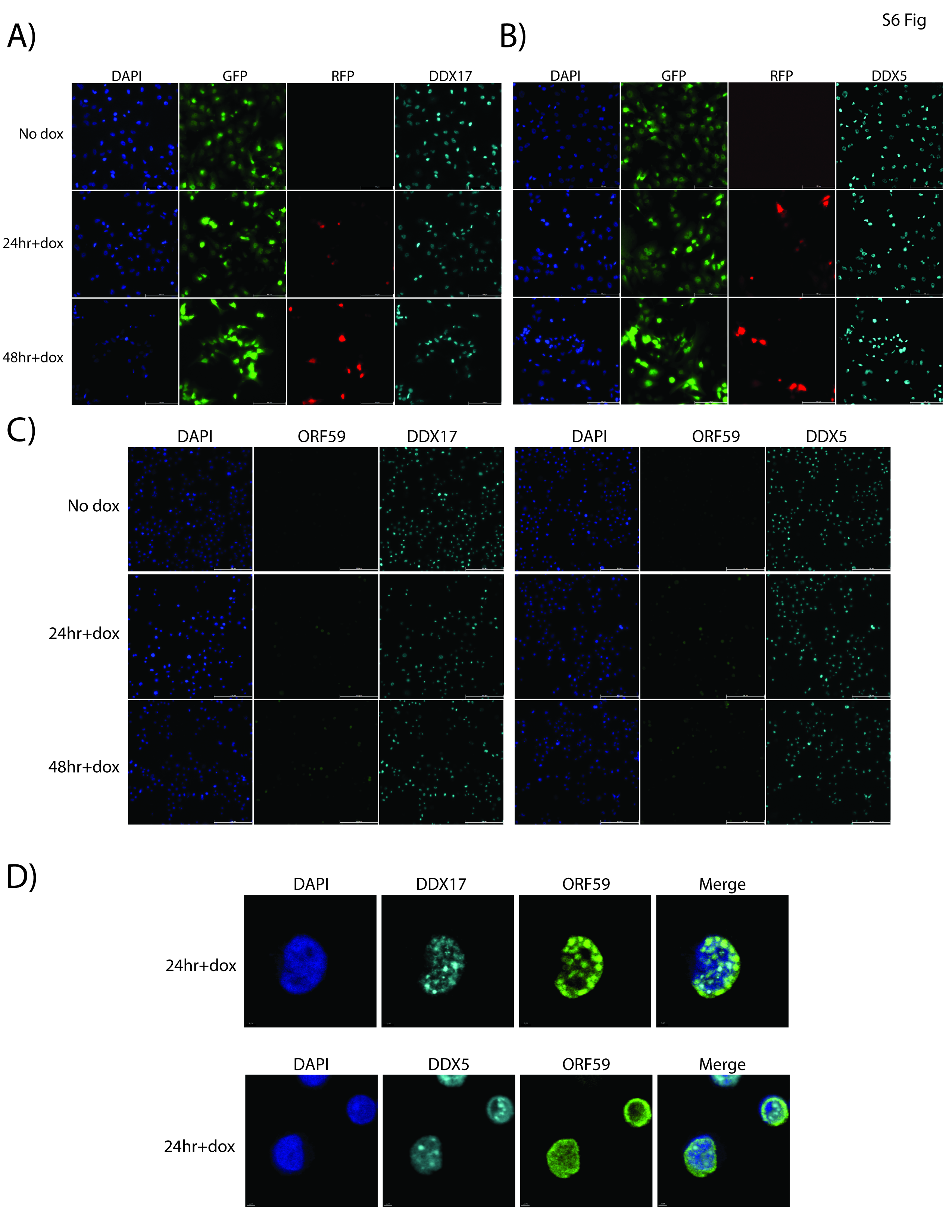

Supplement: S6 Fig — Fluorescent images of A) DAPI, GFP, RFP and DDX17 in iSLK.219 cells, B) DAPI, GFP, RFP and DDX5 in iSLK.219 cells and C) DAPI, ORF59, DDX17 and DDX5 in TREx-BCBL1-RTA cells at 0 h, 24 h, 48 h after doxycycline addition with higher resolution images at 24h post doxycycline in D). (TIF) [file ppat.1013009.s006.tif]

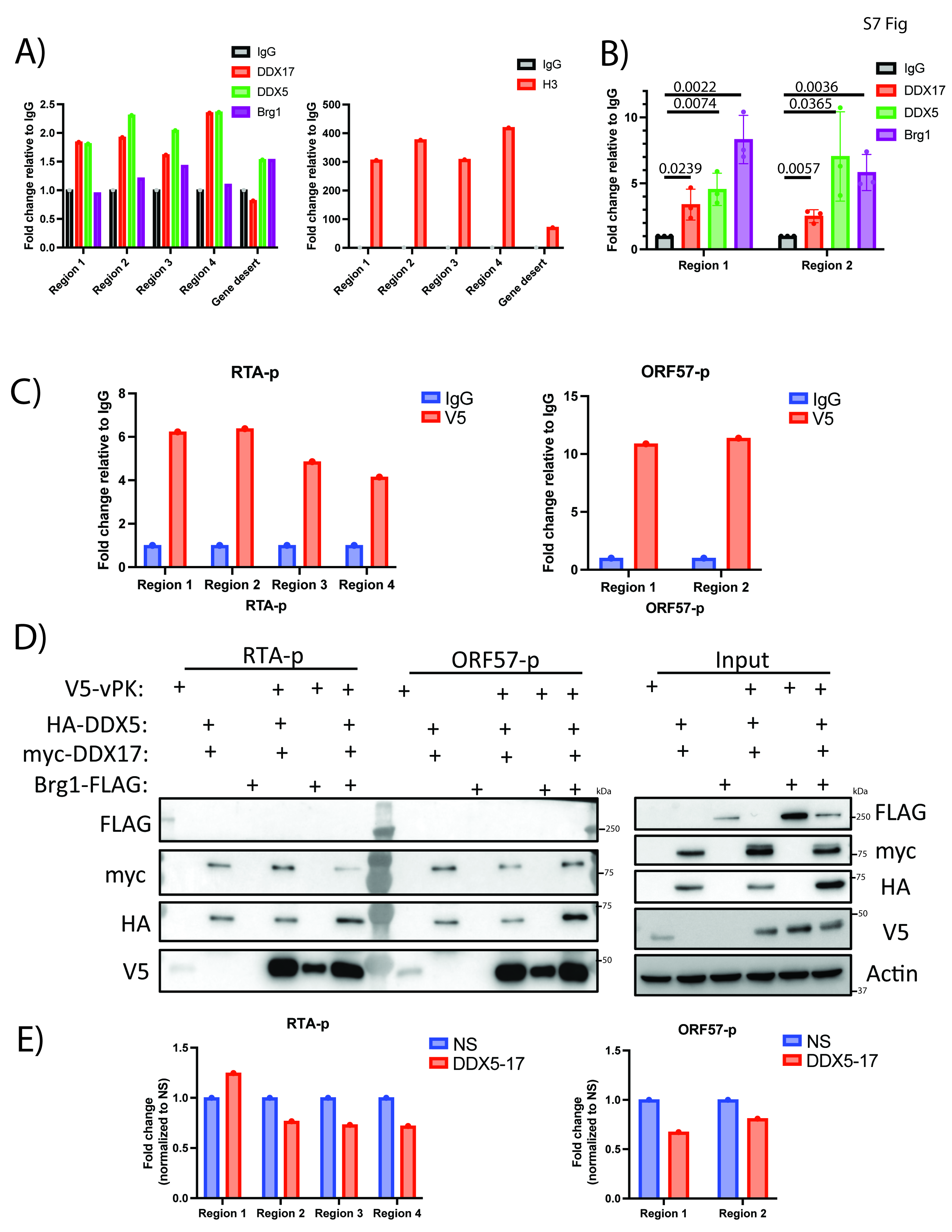

Supplement: S7 Fig — ChIP assay for DDX5, DDX17, Brg1, H3 or control rabbit or mouse IgG in A) latent iSLK.219 cells followed by qPCR with primers targeting 4 regions within the viral RTA promoter and a primer set targeting a gene desert in the human genome as negative control, B) reactivated iSLK.219 cells (24h post doxycycline) followed by qPCR with primers targeting 2 regions of the ORF57 promoter, C) ChIP for V5 or control rabbit IgG followed by qPCR for RTA and ORF57 promoters in 293T.219 cells at 24h post reactivation. D) Western blot for FLAG, myc, HA and V5 after DNA pulldown assay using biotinylated RTA and ORF57 promoters incubated with Brg1-FLAG, myc-DDX17, HA-DDX5 and V5-vPK lysate in the presence of poly dI:dC (10μg/ml). Input blot depicts expression of proteins using actin as a loading control. E) ChIP assay for V5 or control rabbit antibody followed by qPCR for RTA and ORF57 promoters using 293T.219 cells transfected with NS or DDX5-17 siRNAs and overexpressing V5-vPK at 24 h post reactivation, normalized to NS. (TIF) [file ppat.1013009.s007.tif]

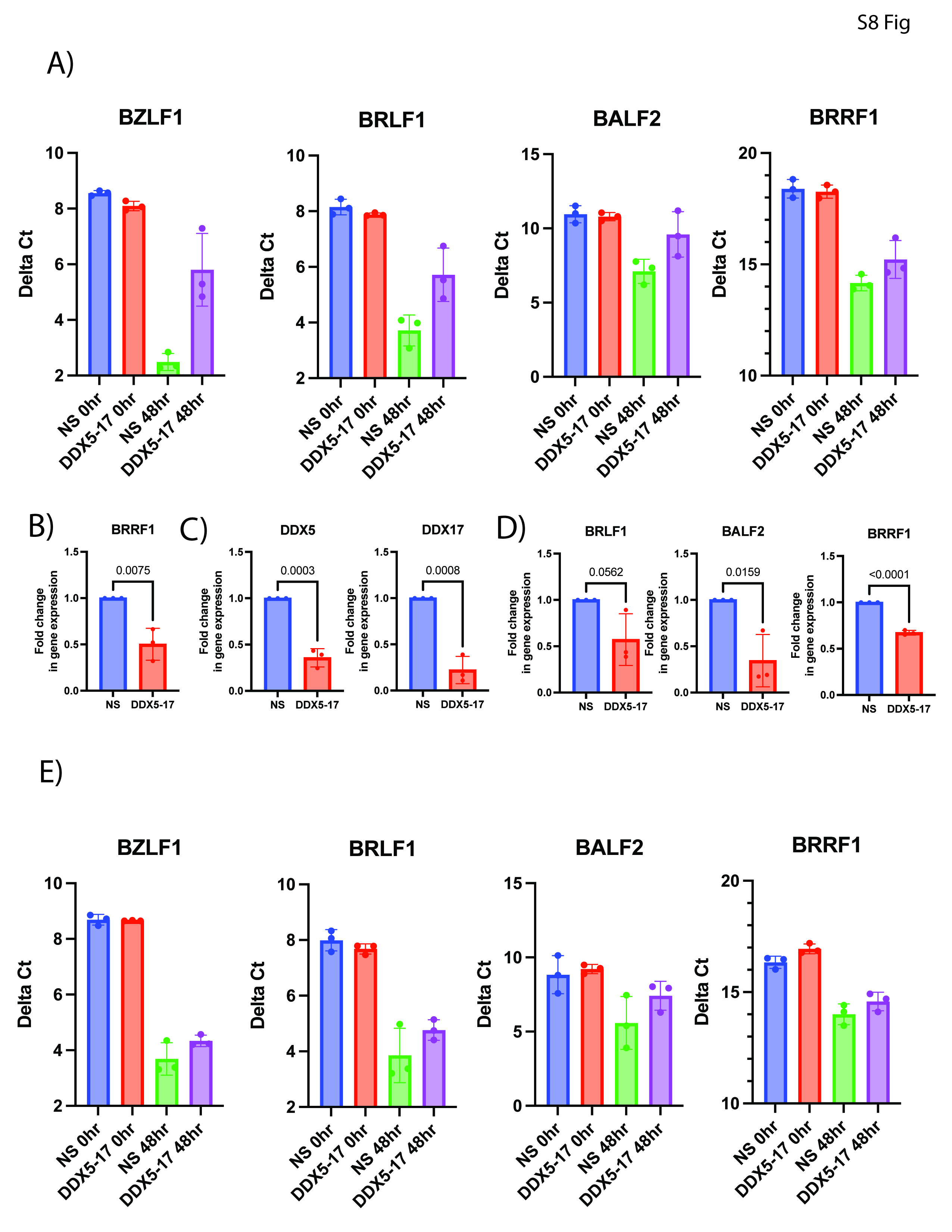

Supplement: S8 Fig — RT-qPCR analysis showing A) Delta Ct of all viral genes in NS or DDX5-17 transfected AGS-EBV cells, B) Fold change of viral gene BRRF1 in NS or DDX5-17 siRNA transfected AGS-EBV cells at 48 h post TPA treatment, C) Fold change of cellular genes DDX5, DDX17 in NS or DDX5-17 siRNA transfected Akata-BX1 cells at 48 h post IgG treatment, D) Fold change of viral genes BRLF1, BALF2 and BRRF1 in NS or DDX5-17 siRNA transfected Akata-BX1 cells at 48 h post IgG treatment with actin as loading control (normalized to NS siRNA) and E) Delta Ct of all viral genes in NS or DDX5-17 transfected Akata-BX1 cells. p values are the result of Student’s t tests and error bars indicate the standard deviation from three independent replicates. (TIF) [file ppat.1013009.s008.tif]

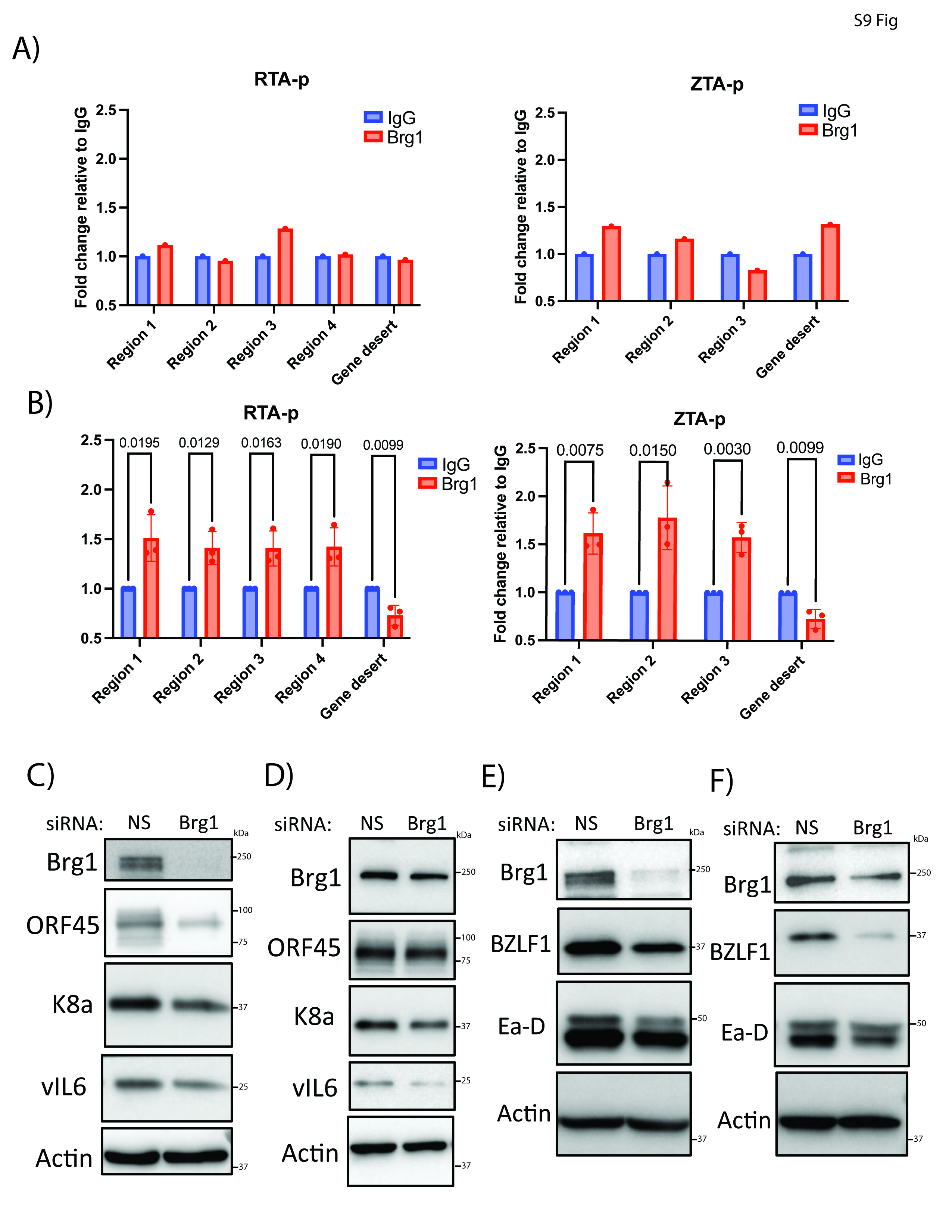

Supplement: S9 Fig — ChIP assay in A) latent AGS-EBV cells (n=1) or B) reactivated AGS-EBV cells (18h post 5ng/ml TPA treatment, n=3) using Brg1 or control mouse antibody and qPCR with primers for 4 different regions for the viral RTA (R) and 3 different regions for the viral ZTA (Z) promoter. Amplification of the gene desert is used as a control. Western blot analysis showing viral protein expression in NS or Brg1 siRNA transfected cells using C) iSLK.219 cells for ORF45, K8a, vIL6 at 24h post doxycycline treatment, D) BCBL1 cells for ORF45, K8a, vIL6 at 24h post VPA treatment, E) AGS-EBV cells for BZLF1 and Ea-D at 48h post TPA treatment and F) Akata BX1 cells for BZLF1 and Ea-D at 48h post IgG treatment. p values are the result of Student’s t tests and error bars indicate the standard deviation from three independent replicates. (TIF) [file ppat.1013009.s009.tif]
