# Supplementary material for: RNA helicases, DDX5 and DDX17, facilitate lytic reactivation of gammaherpesviruses
Source: PLoS Pathog. 2025 Apr 21;21(4):e1013009. doi: 10.1371/journal.ppat.1013009 (PMC12011273; doi:10.1371/journal.ppat.1013009)
Supplement: S1 Table — (DOCX) [file ppat.1013009.s010.docx]

**S1 Table: Supplementary table for primer sequences and siRNA sequences**

**Supplementary table 1:** Primers and siRNA sequences

|  | **qRT-PCR** | **Forward primer 5'-3'** | **Reverse primer 5'-3'** |
| --- | --- | --- | --- |
| KSHV | *vIL6* | CGGTTCACTGCTGGTATCTG | CAGTATCGTTGATGGCTGGT |
| KSHV | *ORF57* | TGGACATTATGAAGGGCATCCTA | CGGGTTCGGACAATTGCT |
| KSHV | *K8.1* | AAAGCGTCCAGGCCACCACAGA | GGCAGAAAATGGCACACGGTTAC |
| KSHV | *RTA* | GAACTGAAGGCCCAACTCTAC | CACACATCTTCCACCACTCTATT |
| Human | *DDX5* | CGGAGGACGGGGTGAAGAT | AAGAAGGTTCTGAACAGCTGGTTAG |
| Human | *DDX17* | GGAAAGGCACCCATCCTTAT | CCTCTGAGCTGTTTGGATAGTC |
| KSHV | *PAN* | GCTCGCTGCTTGCCTTCTT | CCAAAAGCGACGCAATCAA |
| KSHV | *vPK* | TGCGTCCTCTTCCAGTGTTA | GTCAGCAGAGTGTAGCCCAA |
| KSHV | *ORF16* | AGATTTCACAGCACCACCGGTA | CCCCAGTTCATGTTTCCATCGC |
| KSHV | *ORF22* | TCCTCAAGAGTGCCATGTTTAT | GTAGACTATGGGAATGTGCCTATC |
| KSHV | *ORF52* | GATGCCCTGAAGGGACTTT | CCTCTTCGTCGCCTGTTATT |
| KSHV | *ORF42* | AACTGGAGTCTAGTGGAGAGAA | TTCTCCAGGTGCTTGGTAAAG |
| EBV | *BZLF1* | AGGCCAGCTAACTGCCTATC | TGATTCTGGGTTATGTCGGA |
| EBV | *BRLF1* | ACACTCCCGGCTGTAAATTC | TGGCTTGGAAGACTTTCTGA |
| EBV | *BALF2* | GGGCTGTGGCGAGTACCAC | CGCTGGTCCTGTGTGTCTTG |
| EBV | *BRRF1* | AAGAGTCTTGCAGGCTGTAC | GACTAGTAGCTTAGCAGCTCA |
| Human | *Actin* | AAGACCTGTACGCCAACACA | AGTACTTGCGCTCAGGAGGA |
| Human | *47S* | GTGCGTGTCAGGCGTTC | GGGAGAGGAGCAGACGAG |
| Human | *5S* | GGCCATACCACCCTGAACGC | CAGCACCCGGTATTCCCAGG |
| Human | *45S* | CGGGTTATTGCTGACACGC | CAACCTCTCCAGCGACAGG |
| Human | *5.8S* | GAGGCAACCCCCTCTCCTCTT | GAGCCGAGTGATCCACCGCTA |
| Human | *28S* | CTCCGAGACGCGACCTCAGAT | CGGGTCTTCCGTACGCCACAT |

|  | **qPCR for viral genomes** |  |  |
| --- | --- | --- | --- |
| KSHV | ORF39 | GGTTTCCCCTGCTACTTCAA | CATGCTTGGCCCGATATAC |
| EBV | BMRF1 | CAACACCGCACTGGAGAG | GCCTGCTTCACTTTCTTGG |

|  |  |  |  | **Genome coordinates** | |
| --- | --- | --- | --- | --- | --- |
|  | **ChIP-qPCR** |  |  | F' primer | R' primer |
| KSHV | ORF50 (RTA) promoter region 1 | CAAAGGGGTATTGCTCCGGT | CTCCTTGGTGGGTCAGTGTC | 70916-70935 | 70972-70991 |
| KSHV | ORF50 (RTA) promoter region 2 | CCGCCATACTCTTCCAGGAC | AGCACATTACCTCGGACGTG | 70771-70790 | 70818-70837 |
| KSHV | ORF50 (RTA) promoter region 3 | CACCAGGGACGCTAAGAACC | GGTACCACATCGGGTTTCGT | 71316-71335 | 71386-71405 |
| KSHV | ORF50 (RTA) promoter region 4 | CCCCATAGGACCCAGCTACA | GGCTTAATGAGTCGCCGGTA | 71518-71537 | 71566-71585 |
| KSHV | ORF57 promoter region 1 | TTCCATTAGGGTGAGCGAAG | CCACTGGTACCACAAACGAA | 81946-81965 | 82033-82052 |
| KSHV | ORF57 promoter region 2 | GGATAAAGCATACGACACTATATGT | TATGGTTGATGCAGAGGAAGT | 81757-81781 | 81879-81889 |
| EBV | BRLF1 (RTA) promoter region 1 | TCTGGTTTATAGCATCGCGA | CATTAGGCGACTCTGCATCA | 94145-94164 | 93981-94000 |
| EBV | BRLF1 (RTA) promoter region 2 | ACGGCGGCTGGGATTAAATCG | GGTGGTGATGTAGCTATACTT | 94253-94273 | 94173-94193 |
| EBV | BRLF1 (RTA) promoter region 3 | GGAGACCCGTCACGATAGCC | GGCCCACAACTTATGTCTG | 94361-94380 | 94289-94307 |
| EBV | BRLF1 (RTA) promoter region 4 | GACCAACATGTTCAGGAGATT | GGATGTCCAGAGTGCCTACAA | 94509-94529 | 94404-94424 |
| EBV | BZLF1 (ZTA) promoter region 1 | GCCATGCATATTTCAACTGGGCTG | TGCCTGTGGCTCATGCATAGTTTC | 91105-91128 | 91023-91046 |
| EBV | BZLF1 (ZTA) promoter region 2 | GGTGCCCCAGCCACTTGA | GGAATAACAGTGTCAGCCATC | 91421-91438 | 91313-91333 |
| EBV | BZLF1 (ZTA) promoter region 3 | GCGGTGACAGCAGTTCCA | GGTGGACCGCTGCTATCCA | 91908-91925 | 91731-91749 |
| Human | Gene desert chip ctrl | CTAGGAGGGTGGAGGTAGGG | GCCCCAAACAGGAGTAATGA |  |  |

|  | **siRNA sequences** |  |  |
| --- | --- | --- | --- |
| DDX5-17 | GGCUAGAUGUGGAAGAUGUUU |  |  |

|  |  |  |  | **Genome coordinates** | |
| --- | --- | --- | --- | --- | --- |
|  | **Promoter amplification** |  |  | F' primer | R' primer |
| KSHV | KSHV RTA promoter | AATTGGAAGCATTCTCTCTTCAT | TTTTGTGGCTGCCTGGACAGTAT | 68616-68638 | 71672-71694 |
| KSHV | KSHV ORF57 promoter | CACCCAGTATATTTTTTCAAATC | GTCCTTTGGTTCTTATATTGTGC | 81158-81180 | 82146-82168 |
