# Supplementary material for: RNA helicases, DDX5 and DDX17, facilitate lytic reactivation of gammaherpesviruses
Source: PLoS Pathog. 2025 Apr 21;21(4):e1013009. doi: 10.1371/journal.ppat.1013009 (PMC12011273; doi:10.1371/journal.ppat.1013009)
Supplement: S2 Data — (PDF) [file ppat.1013009.s012.pdf]

Fig 1A

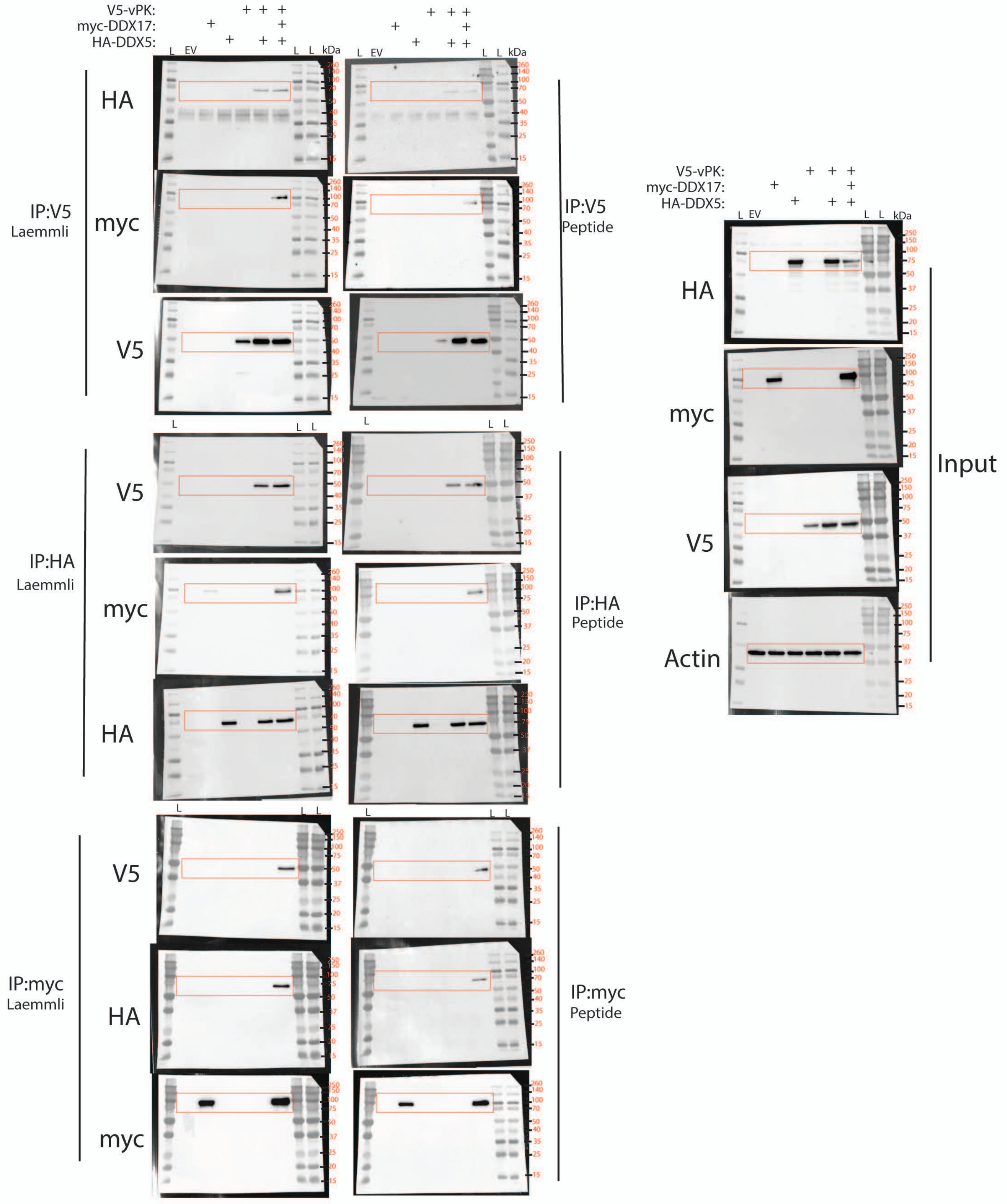

L: Molecular weight ladder
EV: Empty vector
Red boxes highlight the cropped area used in the figure

Fig 1B

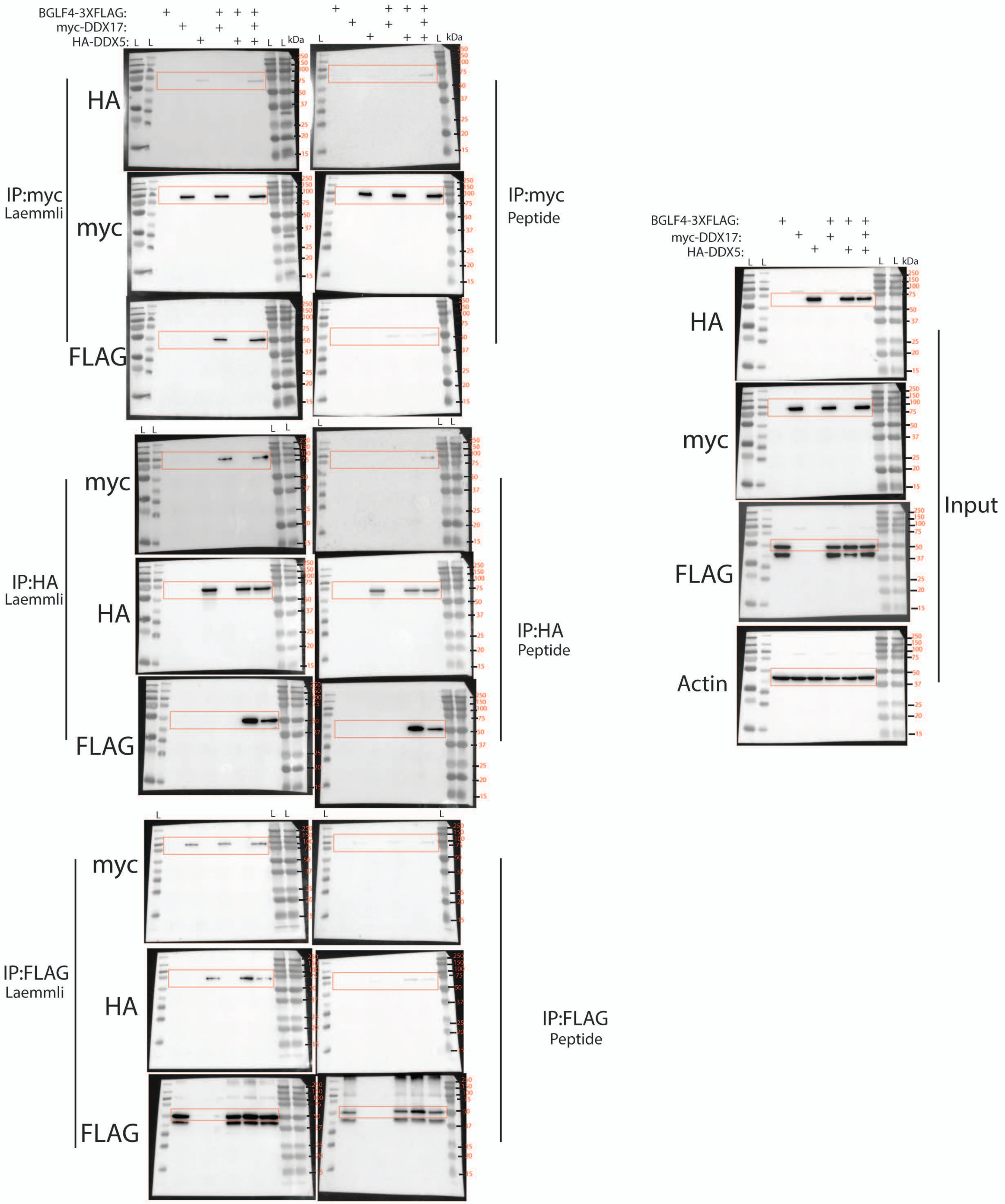

L: Molecular weight ladder
Red boxes highlight the cropped area used in the figure

Fig 2A

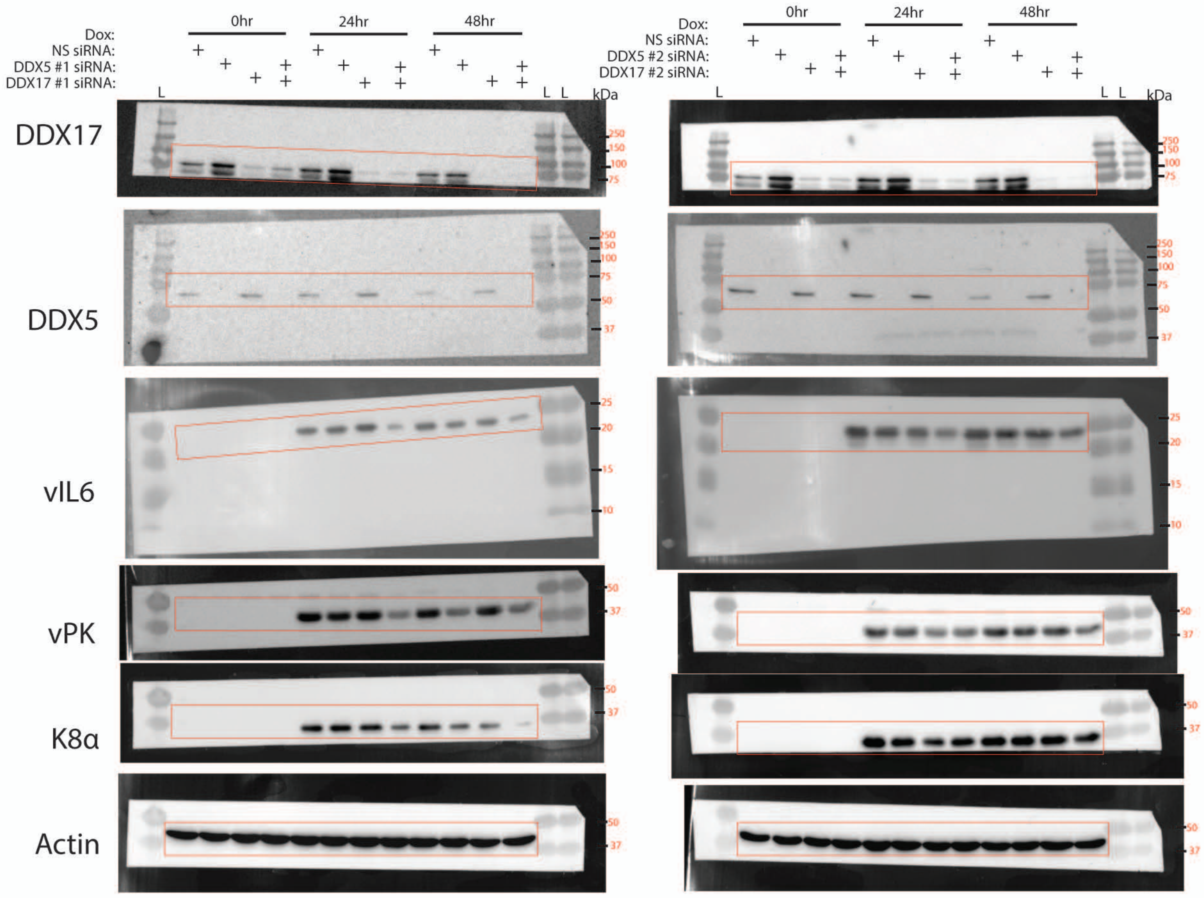

Fig 2B

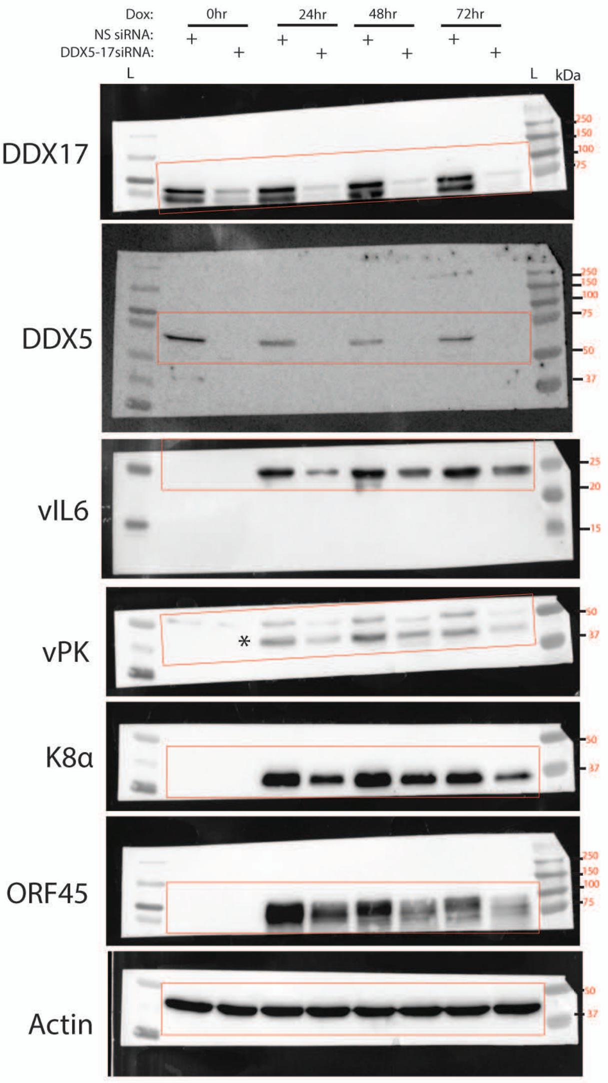

L: Molecular weight ladder
Red boxes highlight the cropped area used in the figure

Fig 4A

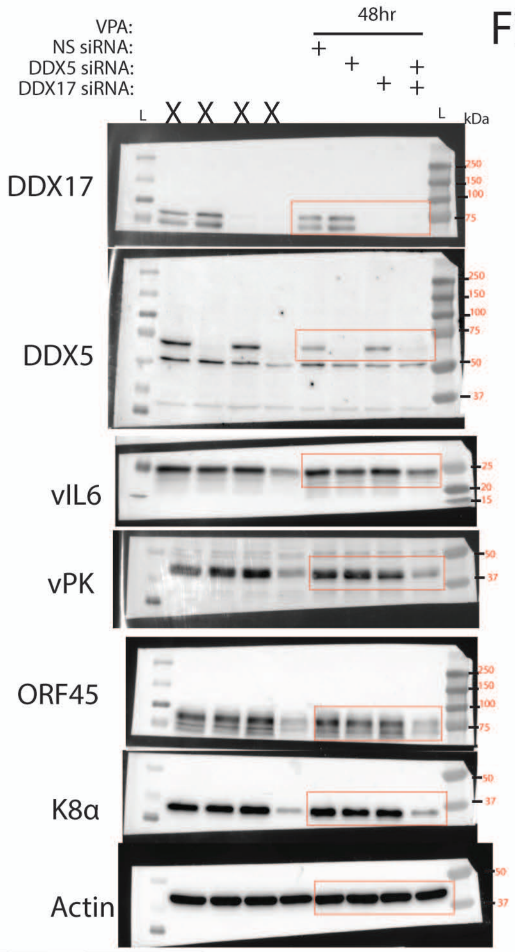

Fig 4B

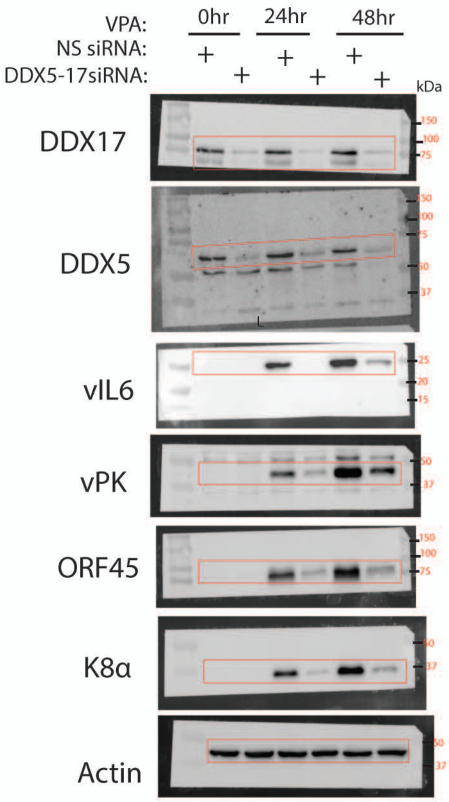

Fig 4E

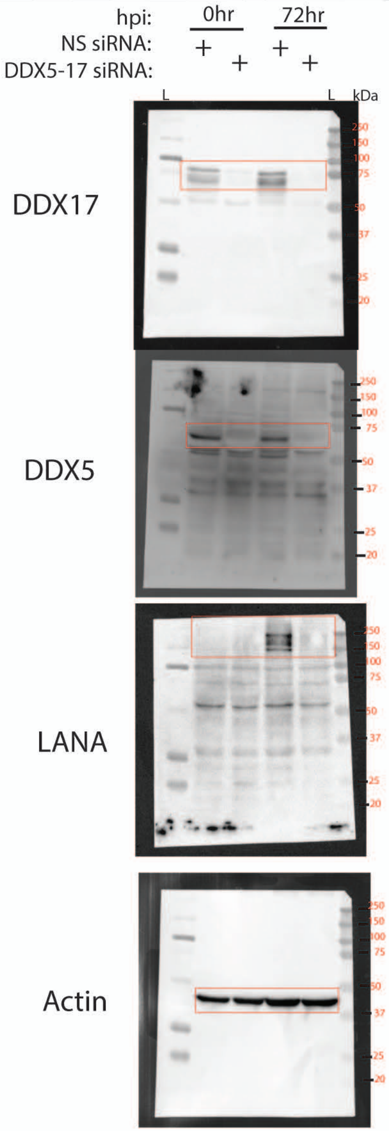

L: Molecular weight ladder
Red boxes highlight the cropped area used in the figure

Fig 5C

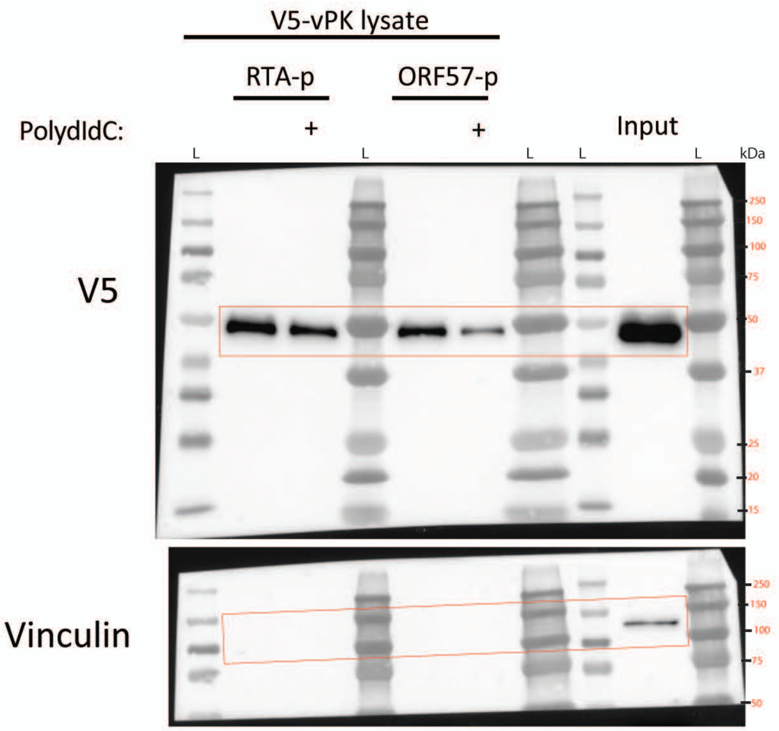

Fig 6A

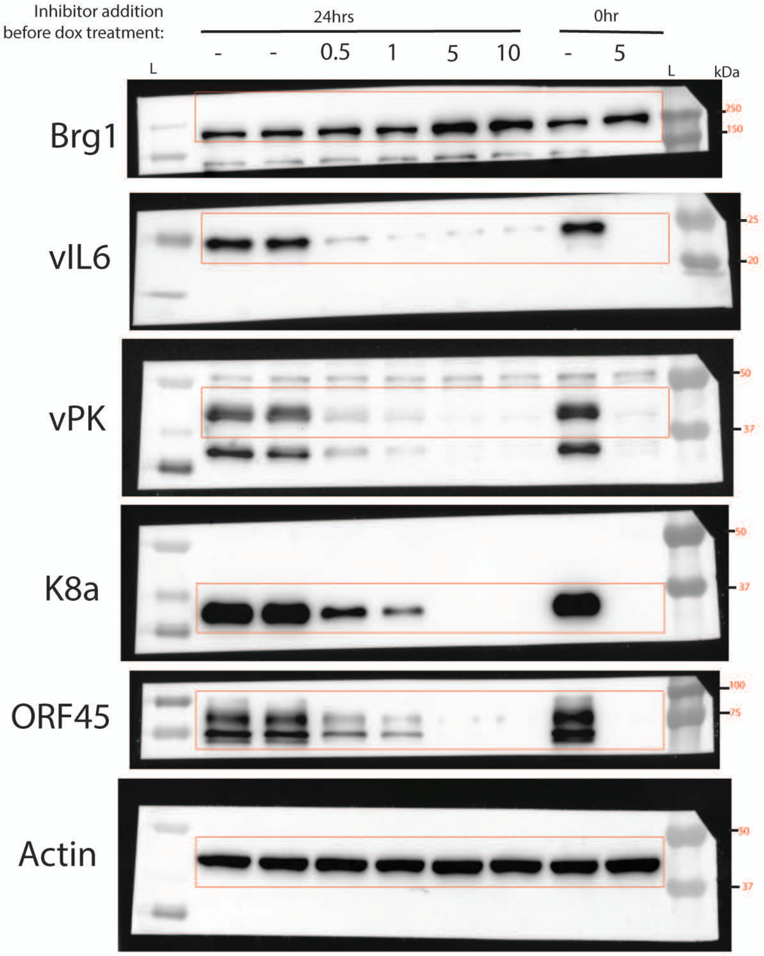

L: Molecular weight ladder
Red boxes highlight the cropped area used in the figure

Fig 6D

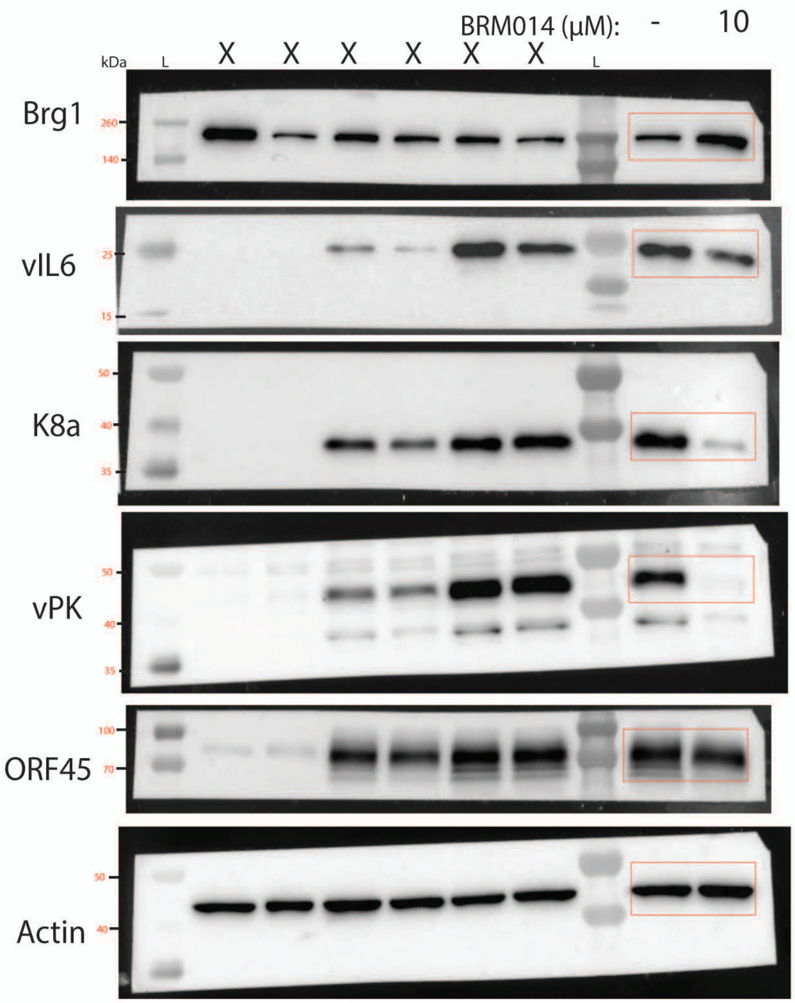

Fig 7A

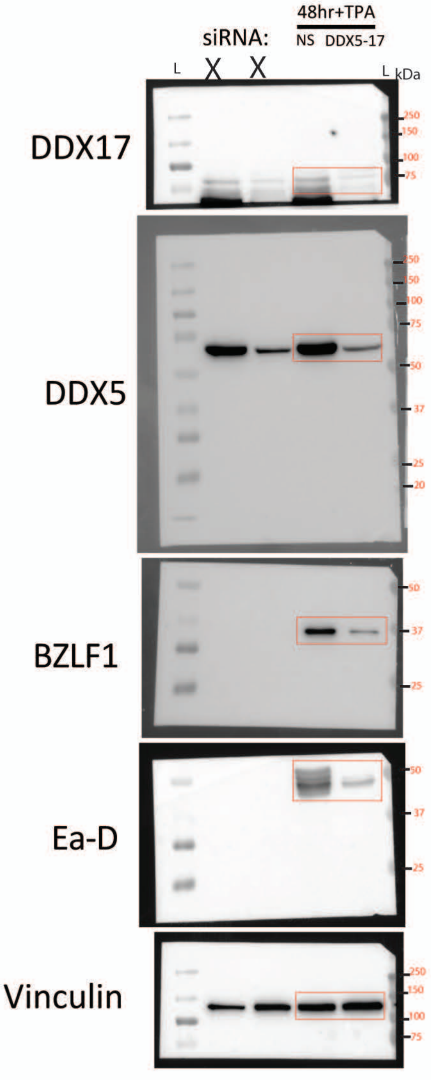

Fig 7E

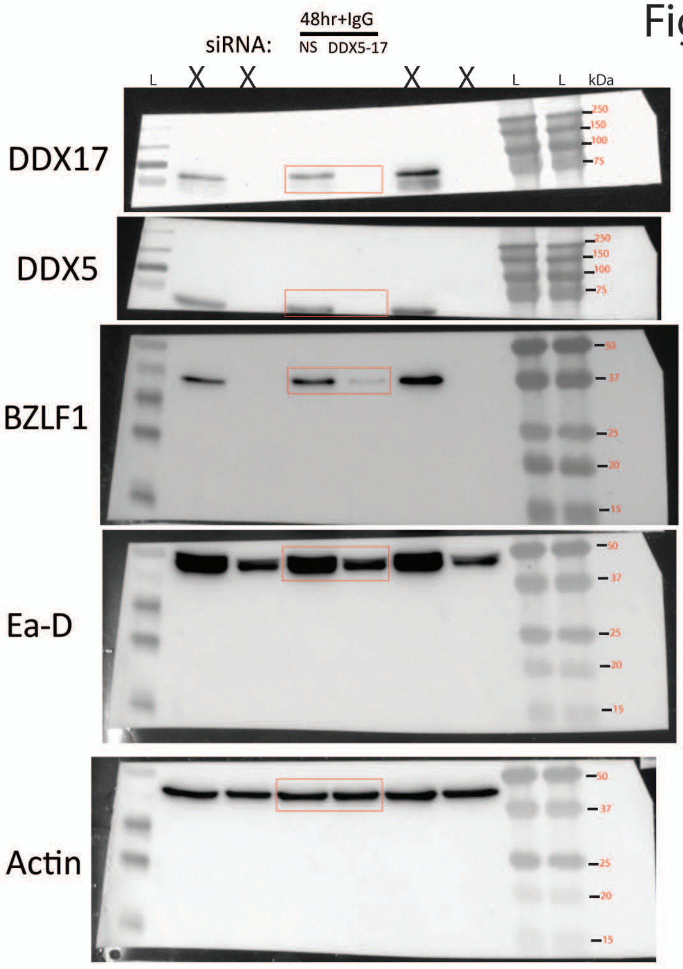

L: Molecular weight ladder

Red boxes highlight the cropped area used in the figure

Fig 8A

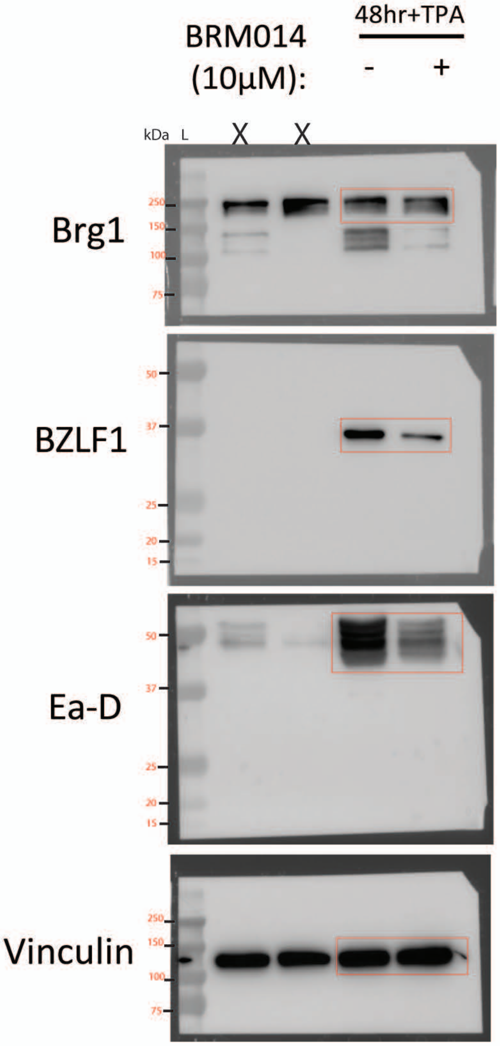

Fig 8D

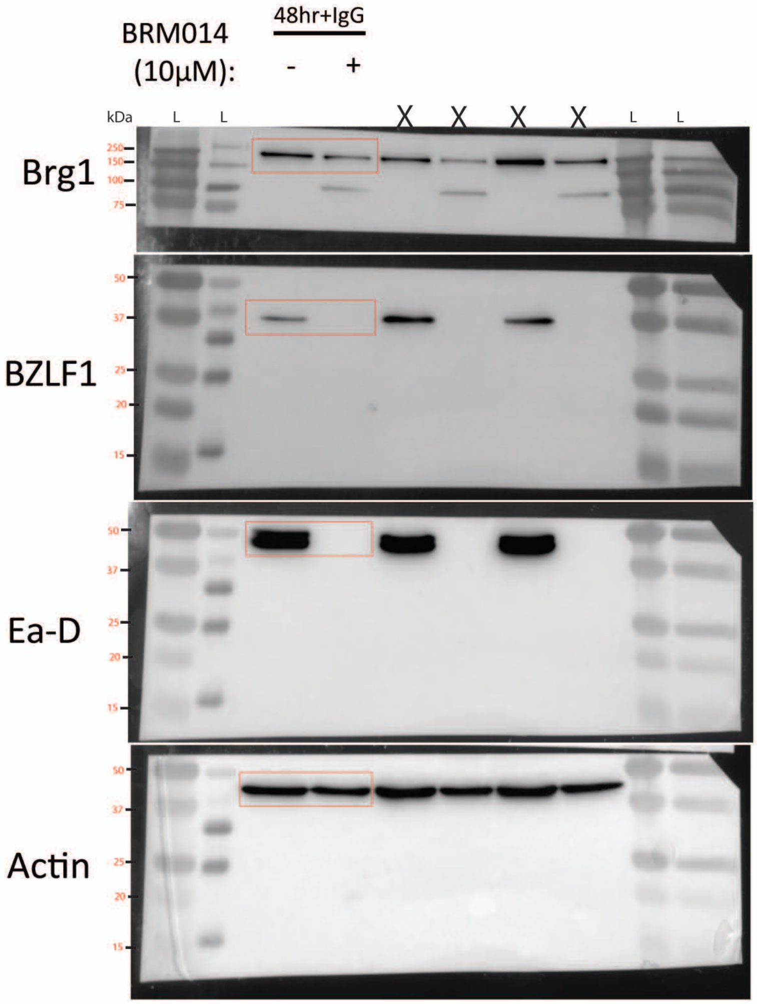

L: Molecular weight ladder
Red boxes highlight the cropped area used in the figure

Fig S1A

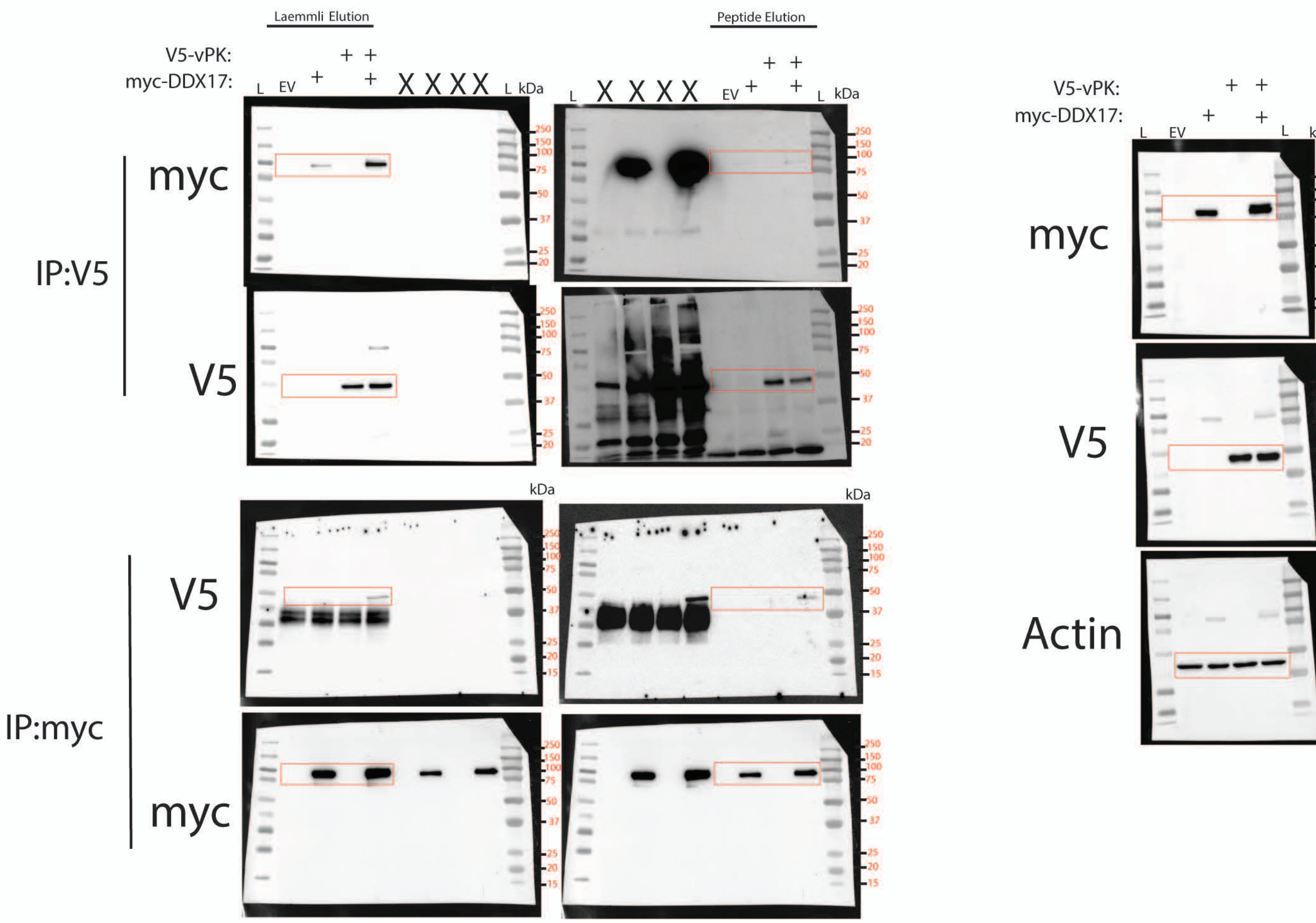

Fig S1B

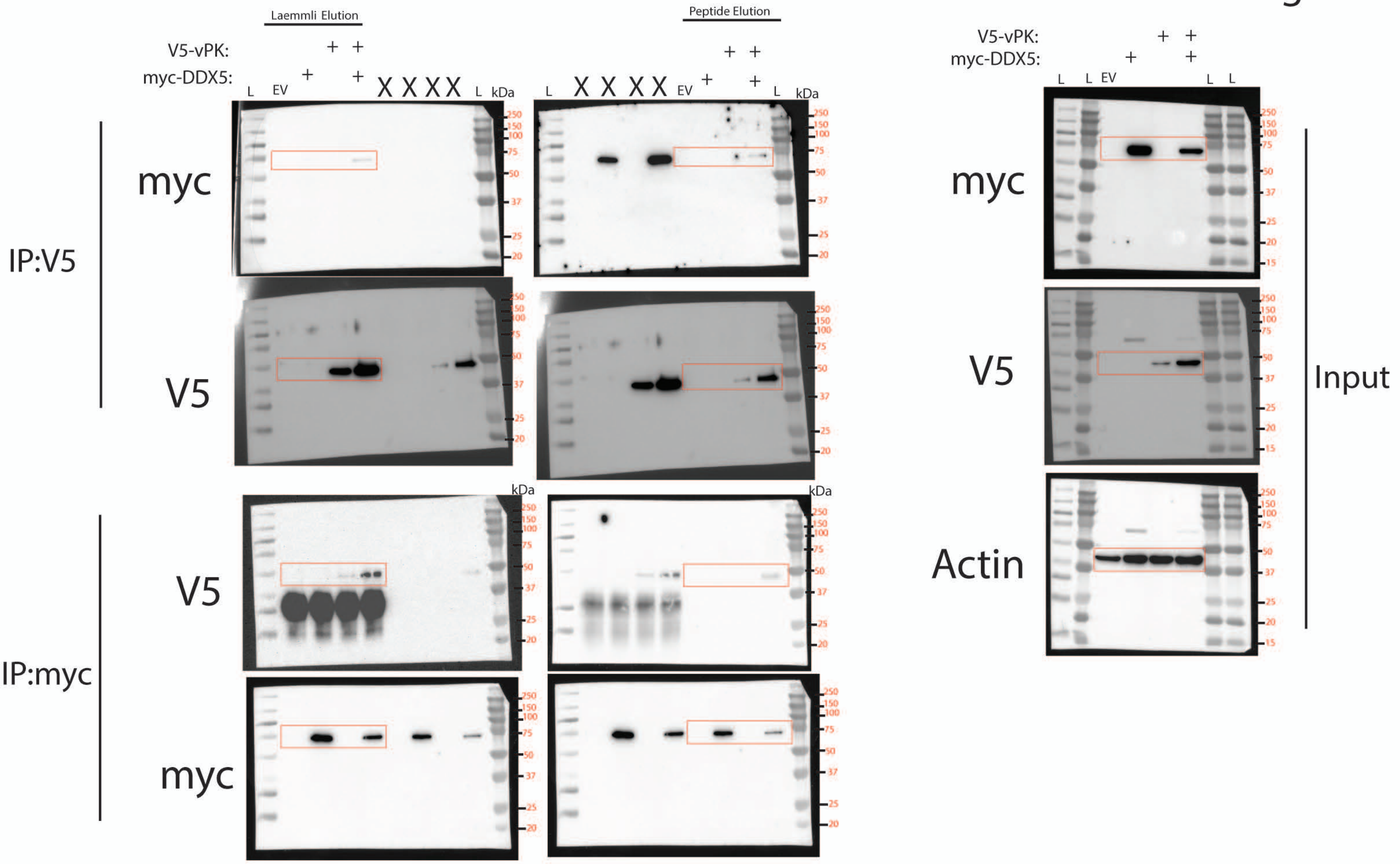

L: Molecular weight ladder
EV: Empty vector
Red boxes highlight the cropped area used in the figure

Fig S1C

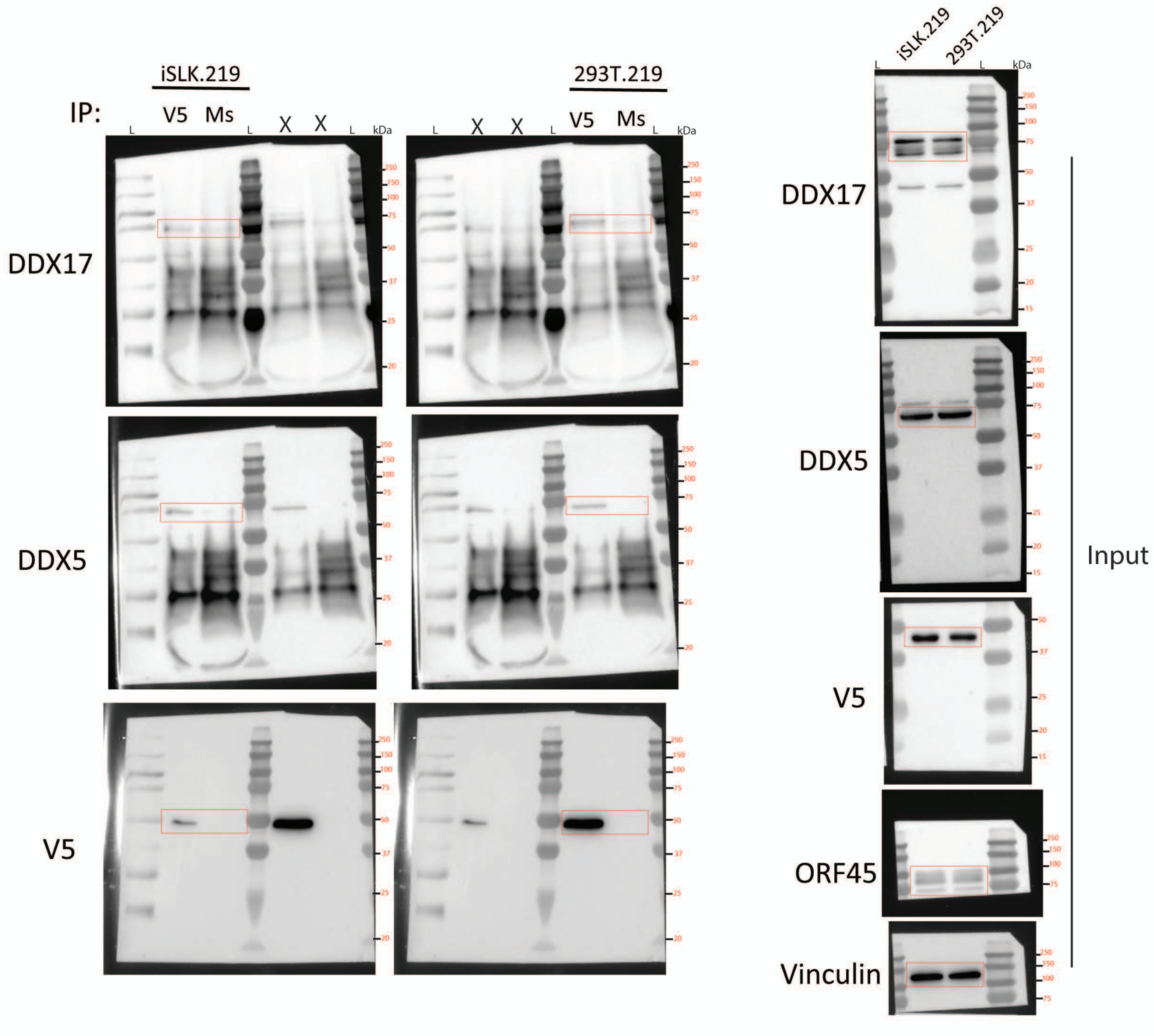

L: Molecular weight ladder
Red boxes highlight the cropped area used in the figure

Fig S7D

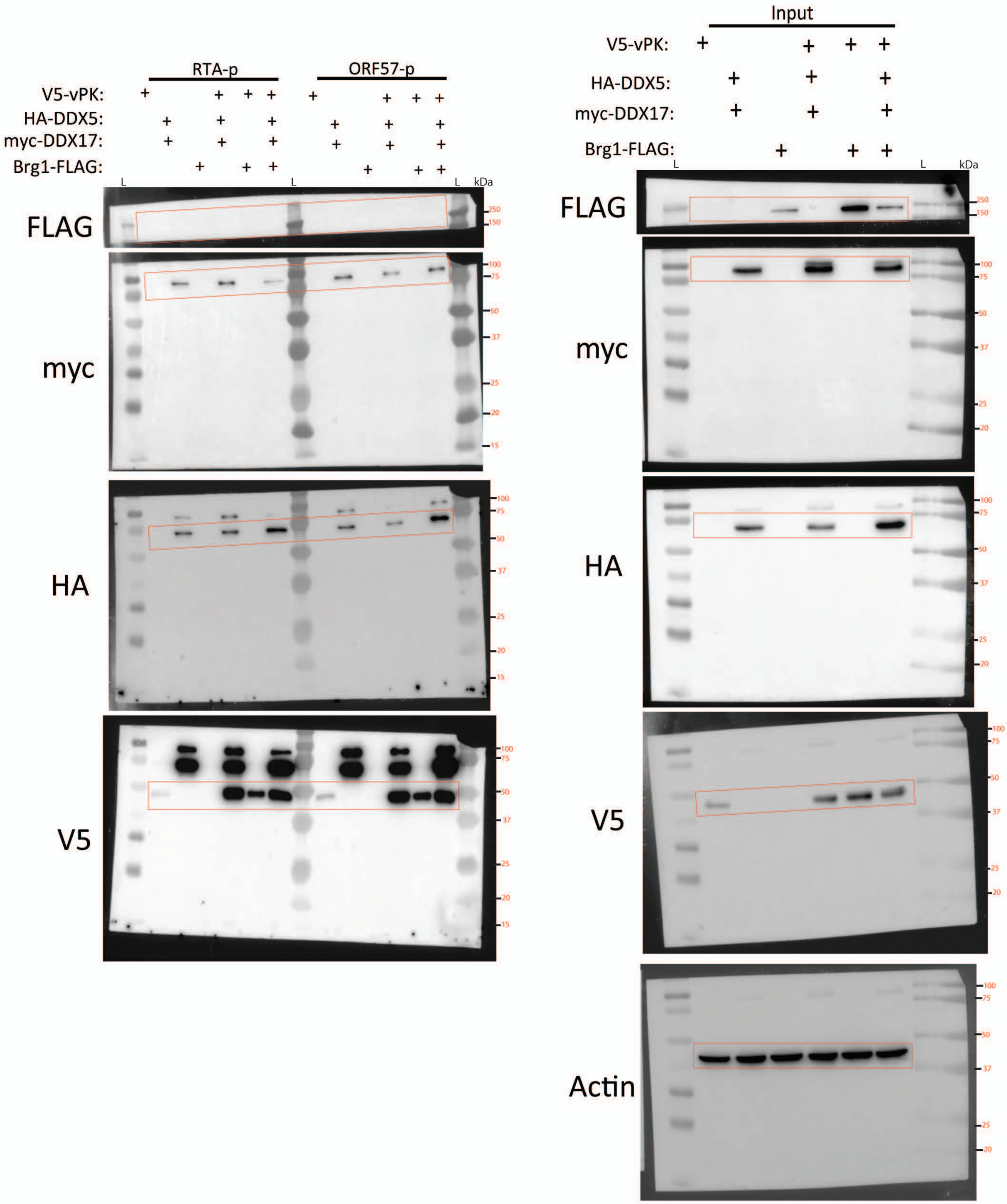

L: Molecular weight ladder
Red boxes highlight the cropped area used in the figure

Fig S9C

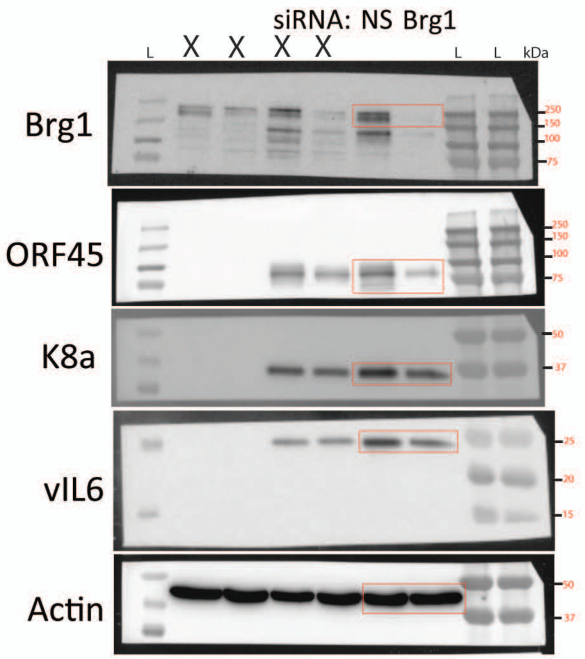

Fig S9D

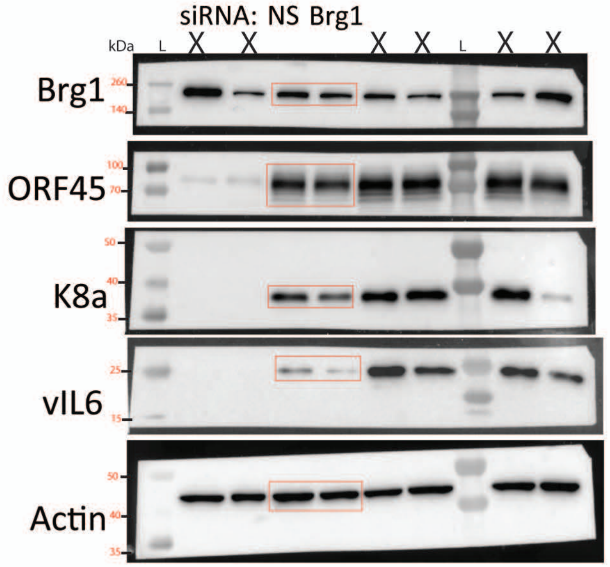

Fig S9E

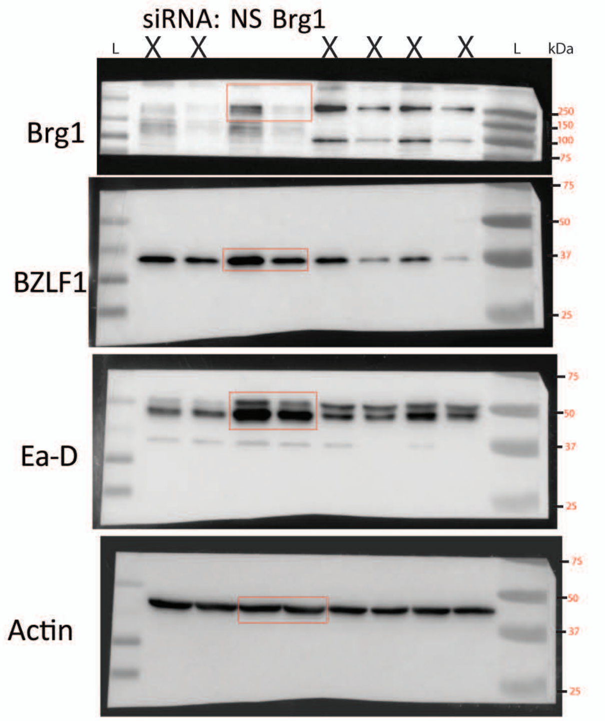

Fig S9F

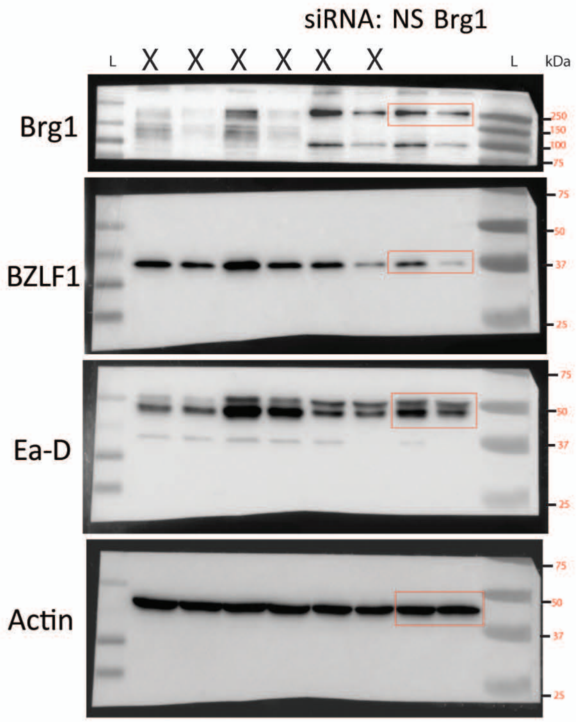

L: Molecular weight ladder

Red boxes highlight the cropped area used in the figure
